# Recent advances in optical sensing strategies for uric acid detection

**DOI:** 10.1039/d6ra07276c

**Published:** 2026-11-02

**Authors:** Suman Nayak, Subhajit Tripathy, Nanjundan Raghul, Priyadip Das, Prasenjit Mahato

**Affiliations:** a Department of Chemistry, SRMIST SRM Nagar, Potheri, Kattankulathur, Kancheepuram District Chennai Tamil Nadu-603203 India priyadipcsmcri@gmail.com priyadip@srmist.edu.in; b Department of Chemistry, Raghunathpur College, Sidho-Kanho-Birsha University Purulia West Bengal-723133 India prasen.mahato@gmail.com

## Abstract

Uric acid (UA), a byproduct of purine metabolism, serves as a significant biomarker associated with various medical conditions, including gout, renal and cardiac disorders, and metabolic dysfunctions. This paper presents a comprehensive review of recent advancements in optical sensing technologies for UA detection. The various platforms are systematically categorized into colorimetric and fluorometric sensors, with particular emphasis on their detection parameters, such as sensitivity, selectivity, and detection limits, as well as their operational principles. Fluorometric sensors are noted for achieving exceptionally low detection thresholds (in the nanomolar range) and high specificity, whereas colorimetric sensors are recognized for their rapid, visual results and reliable performance in complex sample matrices. Numerous international research efforts have contributed to the development of highly sensitive and selective UA detection technologies. Despite the development of several optical chemosensors (both fluorescent and colorimetric) a systematic classification remains absent. This review highlights the design strategies and molecular recognition mechanisms of various UA chemosensors, particularly those based on small organic molecules. The integration of these sensors into wearable technology or diagnostic platforms could significantly advance personalized medicine and the management of UA-related conditions. The paper concludes by outlining potential future directions for the field, including the deployment of multiplexed sensing systems and the application of machine learning for signal analysis, to advance the development of next-generation UA optical sensors for point-of-care diagnostics and real-time health monitoring.

## Abstract 2

Uric acid (UA) is biomarker for gout, kidney, cardiovascular, and metabolic diseases. This review summarizes advances in colorimetric and fluorometric UA sensors, highlighting mechanisms, sensitivity, selectivity, and limits.

## Graphical abstract

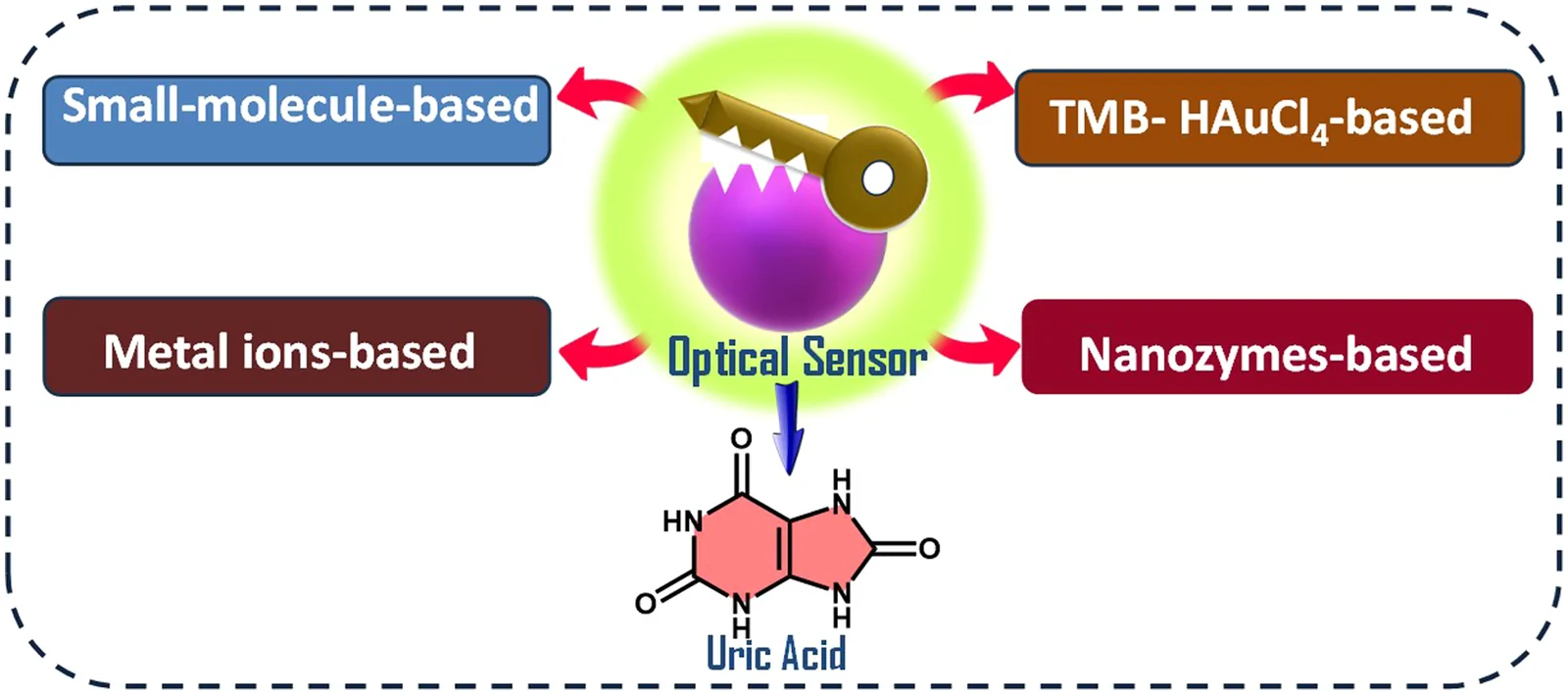

## 1 Introduction

Uric acid (2,6,8-trihydroxypurine, UA) (Fig. 1), the byproduct of purine metabolism, serves as a crucial biomarker in human physiological fluids such as blood serum and urine.^1–3^ The normal concentration ranges for UA in blood serum and urine are 240–520 µM and 1.49–4.46 mM, respectively.^4^ The production of UA, facilitated by xanthine oxidase during purine metabolism, is implicated in various human diseases, including renal disorders, gout, preeclampsia, diabetes, obesity, and cardiovascular diseases.^5–11^ Density Functional Theory (DFT) calculations demonstrate that the triketo tautomer (UA) represents the most thermodynamically stable form in both the gas phase and aqueous solution, with the monohydroxy species UA1 being the subsequent most stable form (Fig. 1).^12^ Furthermore, the deprotonation of uric acid occurs through the loss of a proton from an –OH group on the monohydroxy tautomers, resulting in the formation of the most stable monoanions in an aqueous environment.

**Fig. 1:**
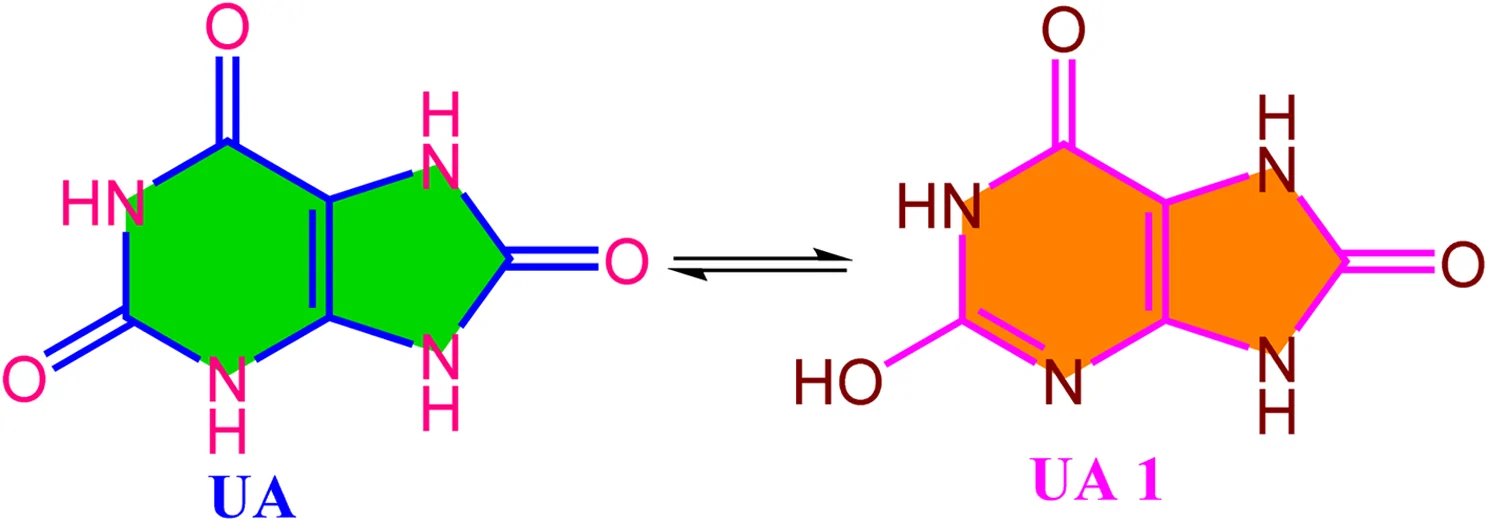
Tautomerisation between the most stable forms of uric acid: the triketo tautomer (UA) and the monohydroxy tautomer (UA1).

UA exists as a urate salt and functions as a weak acid (p*K*_a_ 5.8) at physiological pH; however, excessive accumulation leads to crystallization as monosodium urate (MSU). The balance between UA production and excretion in bodily fluids is maintained within normal limits. Elevated UA levels can precipitate conditions such as preeclampsia, while abnormally low levels may increase the risk of sclerosis.^13^ Consequently, there is a need for a rapid, simple, and cost-effective analytical method for measuring UA levels in clinical samples for disease diagnosis.^14–16^ Recent advancements in UA estimation methods include surface-enhanced Raman spectroscopy (SERS),^13^ capillary electrophoreses with chemiluminescence,^17^ matrix-assisted laser desorption/ionization time of-flight mass spectrometry,^18^ high-performance liquid chromatography (HPLC),^19^ and electrochemical-based methods.^4^ Many of these methods necessitate a complex sample preparation and expensive, sophisticated instrumentation. Therefore, there is a continuous demand for a straightforward, rapid, accurate, and economical approach to UA determination. Colorimetric-based optical techniques^20–24^ and fluorometric sensing^25–30^ systems (chemosensors) have garnered significant interest due to their simple operational procedures and rapid response times. Fluorescence-based sensing devices are favoured for their exceptional sensitivity, selectivity, rapid response, user-friendliness, portability, and relatively low operational costs.^31,32^ This review concentrates on optical sensing techniques for uric acid (UA) detection. Various sensing methods have been employed, including small-molecule fluorescent chemosensors, nanostructures, silver nanoprisms (utilizing surface plasmon resonance), metal–organic frameworks (MOFs), nitrogen-doped carbon dots with chromium(vi), coordination polymers, and others for the selective identification of UA. The review will elucidate the rationale behind sensor design, detection limits for the analyte, selectivity for UA, and their potential biological applications. The primary challenges in developing an optical sensor for UA are highlighted in this review article, along with possible solutions. Additionally, potential advancements and future trends in this field are explored, paving the way for further research and innovation.

## 2 Chemosensors for uric acid (UA)

Designing optical chemosensors for the recognition of uric acid entails the development of molecular or material-based systems capable of selectively interacting with uric acid and eliciting a measurable alteration in optical signals, such as color or fluorescence. Uric acid is a crucial biomarker for various diseases including gout, kidney dysfunction, and metabolic disorders, so its precise detection is essential in clinical diagnostics. The literature presents a variety of optical chemosensors for uric acid, which are broadly categorized under specific headings and will be discussed subsequently.
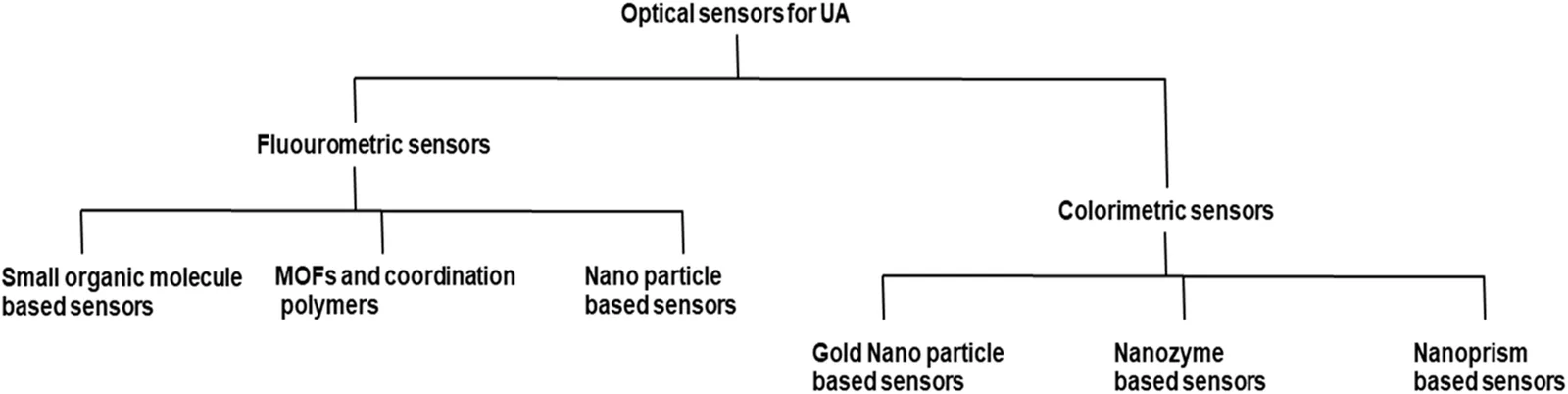


### 2.1 Fluorescent chemosensors for UA recognition

In recent years, fluorescent chemosensors have emerged as a compelling alternative due to their exceptional sensitivity, selectivity, user-friendly operation, and applicability for real-time, non-invasive monitoring. These chemosensors alter their photophysical properties, such as emission wavelength or intensity, in response to specific interactions with the target analyte (Fig. 2). The recognition unit of the chemosensor preferentially interacts with uric acid molecules, often through electrostatic interactions, π–π stacking, or hydrogen bonding. Various researchers have utilized, metal–organic frameworks (MOF) as luminescent sensor for the recognition of UA. Additionally, nanoparticle-based luminescent chemosensors capable of detecting UA by quenching emission signal of nanoparticles have also been documented. Through the action of uricase, uric acid is converted into allantoin with the concurrent formation of H_2_O_2_, which subsequently quenches the emission of the luminescent nanoparticle (Fig. 1). Here, we have provided a schematic illustration of several widely used techniques that have been applied to the fluorescence detection of UA (Fig. 2).

**Fig. 2:**
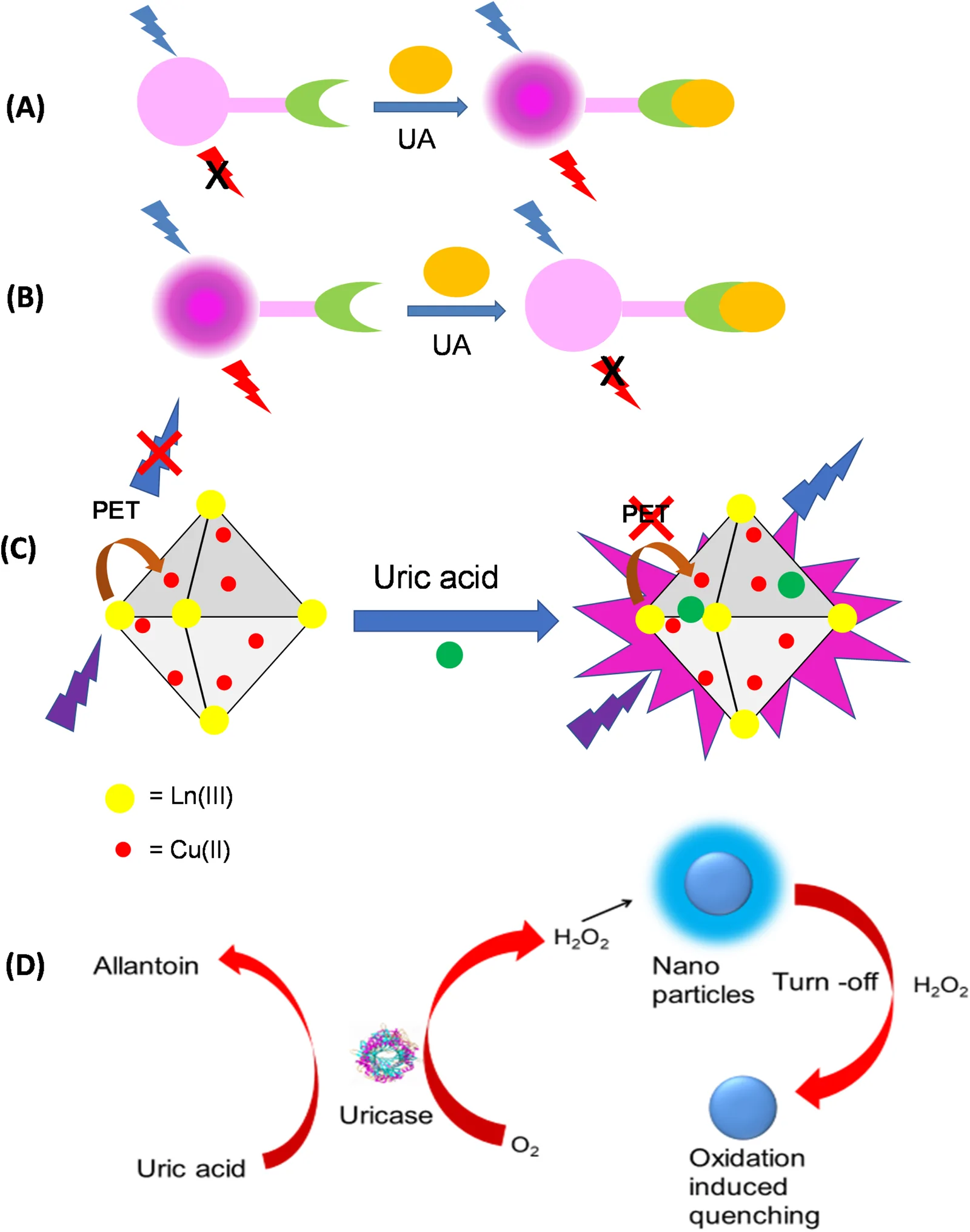
(A) Schematic illustration of a molecular luminescent sensor, whose emission turned-off upon UA recognition. (B) Schematic illustration a molecular luminescent sensor, whose emission turned-on upon UA recognition. (C) Schematic illustration of UA recognition by MOF based fluorescent sensor, where emission is turned-on upon UA binding due to the inhibition of photoinduced electron transfer (PET) process. (D) Schematic representation of UA recognition by nanoparticle-based fluorescent sensor.

#### 2.1.1 Small-molecule based fluorescent chemosensors for UA recognition

In their endeavour to develop a fluorogenic chemosensor for UA, the Dey group engineered a novel rhodamine–napthyridine-based probe (1; Fig. 3).^33^ The probe was synthesized through a condensation reaction between Rhodamine B and 7-chloro-1,8-napthyridine-2-amine. The structure and geometry of compound 1 were confirmed through single-crystal X-ray crystallography. Upon introduction of the analyte into the probe solution, a distinct color change from colorless to pink was observed, attributed to the opening of the rhodamine ring. Concurrently, a new fluorescence emission peak appeared at 561 nm resulting from the ring-opening process and strong hydrogen bonding between the probe and UA. The probe's advantage lies in its capacity to specifically and selectively detect UA through a visually discernible luminescent response under UV light, exhibiting a pronounced “off-on” emission. Cytotoxicity assays demonstrated that the probe is non-toxic to live cells, suggesting its potential for diagnostic applications in human health. Additionally, probe 1 was employed for *in vitro* UA detection in live HeLa cells. Using DFT calculations, the authors also elucidated the probable binding mode and optimized geometry of 1 and its complex with UA. They proposed that the probe could be further applied for *in vivo* detection of UA in the human body.

**Fig. 3:**
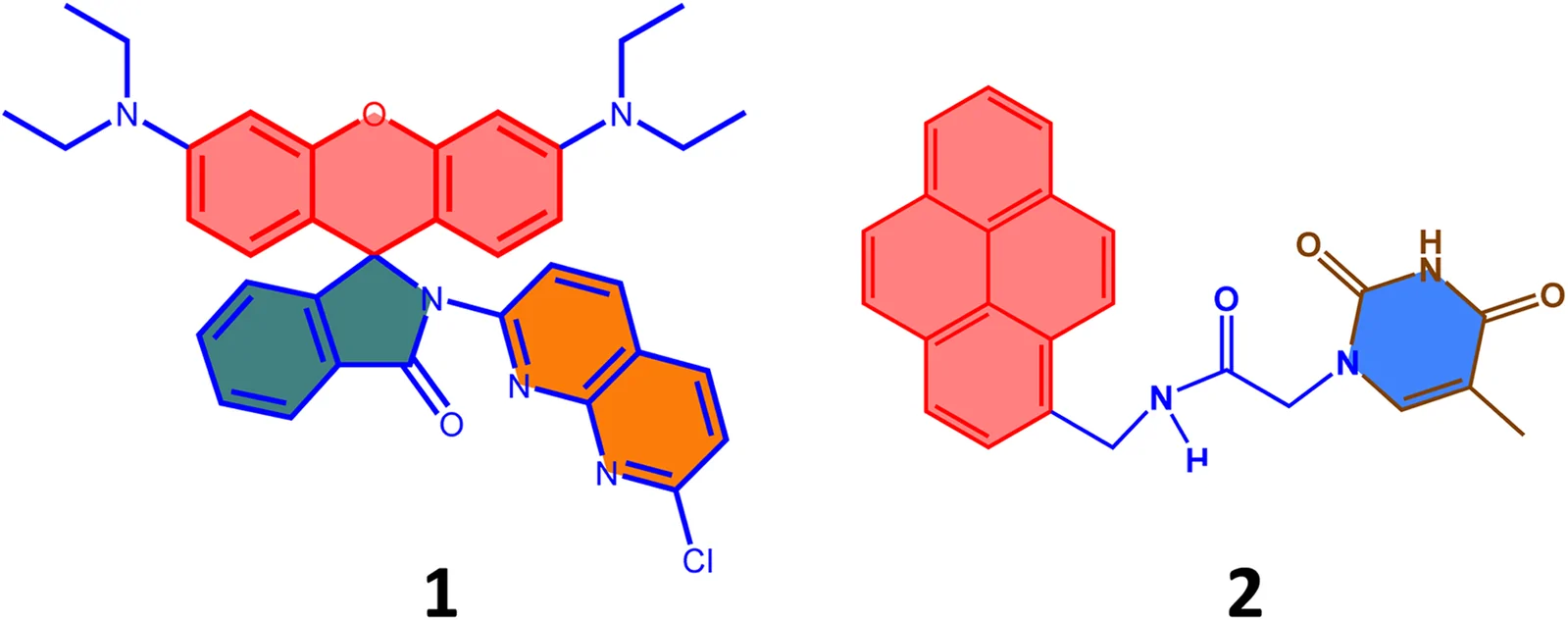
Schematic representation of the fluorescent chemosensor 1 and 2.

The Sahoo group has developed a novel pyrene-appended thymine derivative (2; Fig. 3) for the selective fluorometric detection of UA in aqueous media.^34^ The fluorescent pyrene moiety facilitates π-stacking interaction with the guest unit, while the thymine acetamide component of the probe provides hydrogen bond donor acceptor groups for favorable complexation. To verify the interaction between compound 2 and UA, spectrophotometric and spectrofluorometric titrations were conducted by introducing varying concentrations of the guest to a fixed concentration of the receptor. As the concentration of UA increased, the absorption spectra of PTA recorded in an aqueous medium (10 mM HEPES buffer, pH 7.4) exhibited a gradual enhancement of the band at 292 nm, while the bands at 330 nm and 346 nm experienced a slight hypsochromic shift accompanied by reduced intensity. Upon the addition of the guest up to two equivalents, the emission peaks of the probe at 377 nm and 395 nm (*λ*_ex_ = 315 nm) progressively increased, as indicated by emission spectroscopy. Titrimetric results revealed an association constant of *K*_a_ = 3.10 × 10^4^ M^−1^, confirming a 1 : 2 stoichiometry between the host and the guest. The sensor exhibited selective detection of UA amidst biologically relevant purine bases, as adenine, guanine, caffeine, theophylline, and hypoxanthine.

Several biologically significant purine bases were employed to evaluate sensor's selectivity, revealing that only the presence of UA elicited a turn-on response. The host–guest complexation in the presence of water molecules was further examined through quantum mechanical computation at the TDDFT level utilizing the B3LYP/6-31+G(d,p) level of theory. Investigations demonstrated that the HOMO of the PTA–UA complex was predominantly delocalized across the whole π-conjugated –NH–CO structure, including the pyrene moiety, while the LUMO was largely concentrated on UA. In confocal microscopy studies, cells incubated with UA exhibited enhanced blue fluorescence. The group reported that the probe is uniquely capable of recognizing UA in live cells.

For the selective recognition of uric acid in live-cell environments, the Sahoo group introduced a novel naphthyridine-based fluorescent probe 3 (Fig. 4).^35^ Due to its exceptional chemical stability and high emission quantum yield, the naphthyridine moiety was chosen as the signaling component. The UV-vis absorption spectra in a water–DMSO (9 : 1, v/v) mixture with a 10 mM HEPES buffer at pH 7.4 demonstrated a decrease in absorption intensity at 285 nm and an increase at 326 nm as UA was incrementally added. The formation of a host–guest complex was confirmed by a distinct isosbestic point at 313 nm. Emission spectroscopy results (Fig. 4B) indicated that the fluorescence emissions of the probe at 355 nm and 420 nm were significantly quenched in the presence of UA. Through UV-vis absorption and emission titrations, a 1 : 1 adduct formation between the host and guest was verified. The probe's high sensitivity allowed for the detection of UA concentrations as low as 0.6 µM. DFT-TDDFT calculations were conducted to examine the electronic characteristics of the host–guest complex and the sensing mechanism. To validate the proposed recognition mechanism, the group performed ^1^H NMR titration of the host and guest in a DMSO-d^6^–D_2_O medium. Confocal microscopy images demonstrated that the bright blue emission of probe 3 was in the presence of UA in Vero 76 cells (Fig. 4C). Furthermore, probe 3 exhibited the capability to measure UA levels in urine and blood serum. According to the researches, this probe holds significant potential as a tool for investigating the role of UA in biological and clinical processes.

**Fig. 4:**
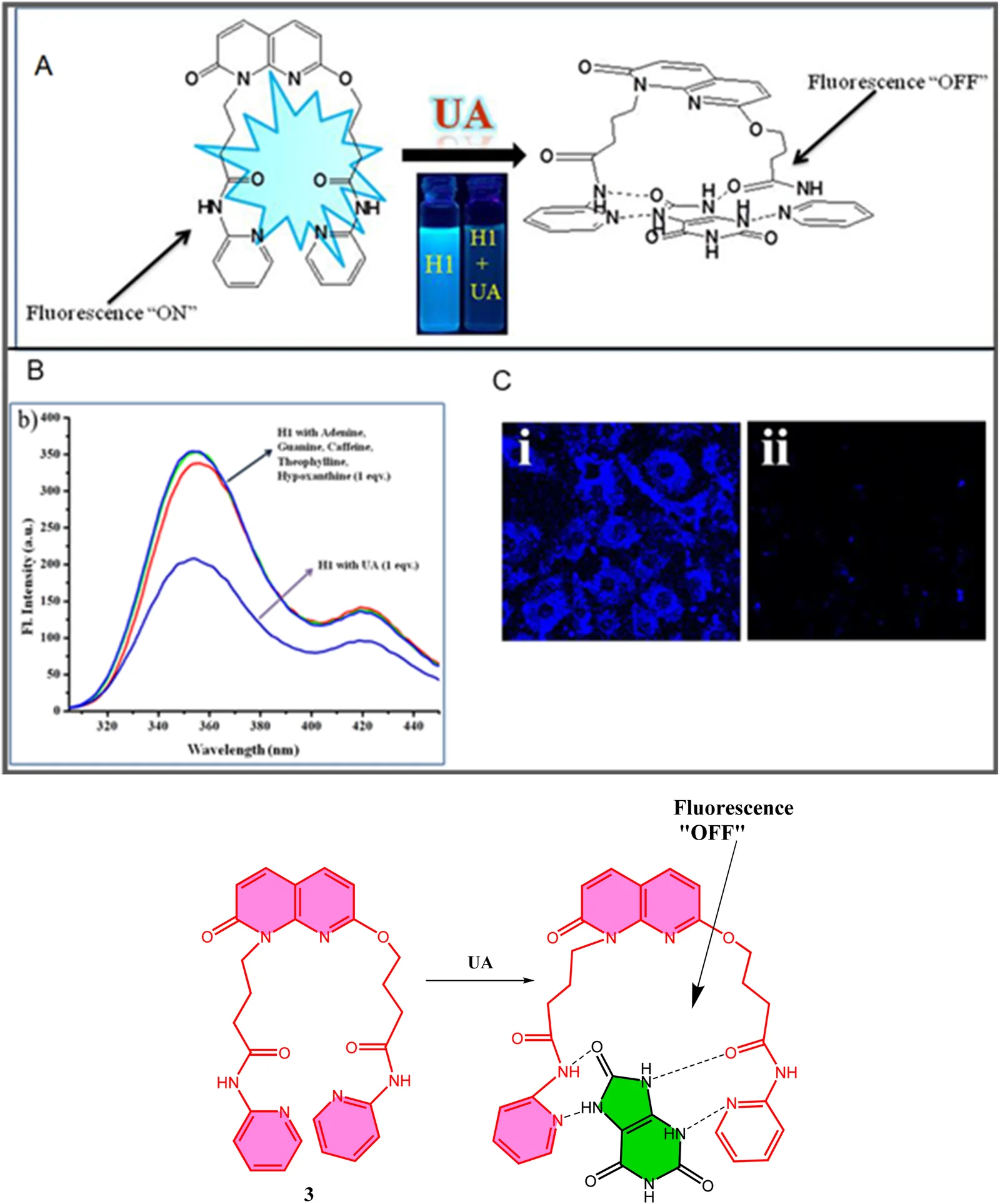
(A) Schematic representation of the fluorescent detection of UA by the chemosensor 3. (B) Emission spectra (*λ*_ex_ = 285 nm) of 3 with 1 equiv. of UA and others purine derivatives. (C) Confocal microscopic images of Vero 76 cells with (i) 3 (2.0 × 10^−5^ M) and (ii) 3 (2.0 × 10^−5^ M) + UA (1.0 × 10^−4^ M) (this figure has been adapted from ref. 35 with permission from Elsevier, copyright 2017).

Elevated concentrations of acid (UA) are associated with various health disorders. To facilitate a cost-effective and rapid detection of UA, Akinyode and colleagues developed a naphthalimide-based “turn-on” chemosensor.^36^ This sensor was subsequently utilized in the development of a test paper strip. The chemosensor was immobilized onto cellulose paper by using divinyl sulfone (4; Fig. 5). The design of the chemosensor was predicated on the concept that an effective photoinduced electron transfer (PET) chemosensor comprises two distinct moieties: a binding moiety and a signaling moiety. To inhibit PET and activate emission from the naphthalimide moiety, thereby confirming the identification process, 1-(2-propynyl) piperazine formed a H-bond with the analyte. The sensor was immobilized on the cellulose surface using an Epson R280 inkjet printer. A solution comprising 10% w/v naphthalimide sensor and 5% w/v TWEEN 20 in 50% acetonitrile/0.1 M pH 10 sodium carbonate buffer was prepared for the printer. The chemosensor modified test strips were immersed in solution containing varying concentration of uric acid and a calibration curve was established. Furthermore, authors demonstrated the capability to detect UA in grain and synthetic urine samples. According to the calibration curve results, grain samples contained UA within the calibration curve's range, in contrast, synthetic urine exhibited UA levels slightly below the calibration curve, likely due to quenching of the probe in urine. The emission of 4 is activated in the presence of uric acid, whereas it is quenched in the absence of the uric acid analyte.

**Fig. 5:**
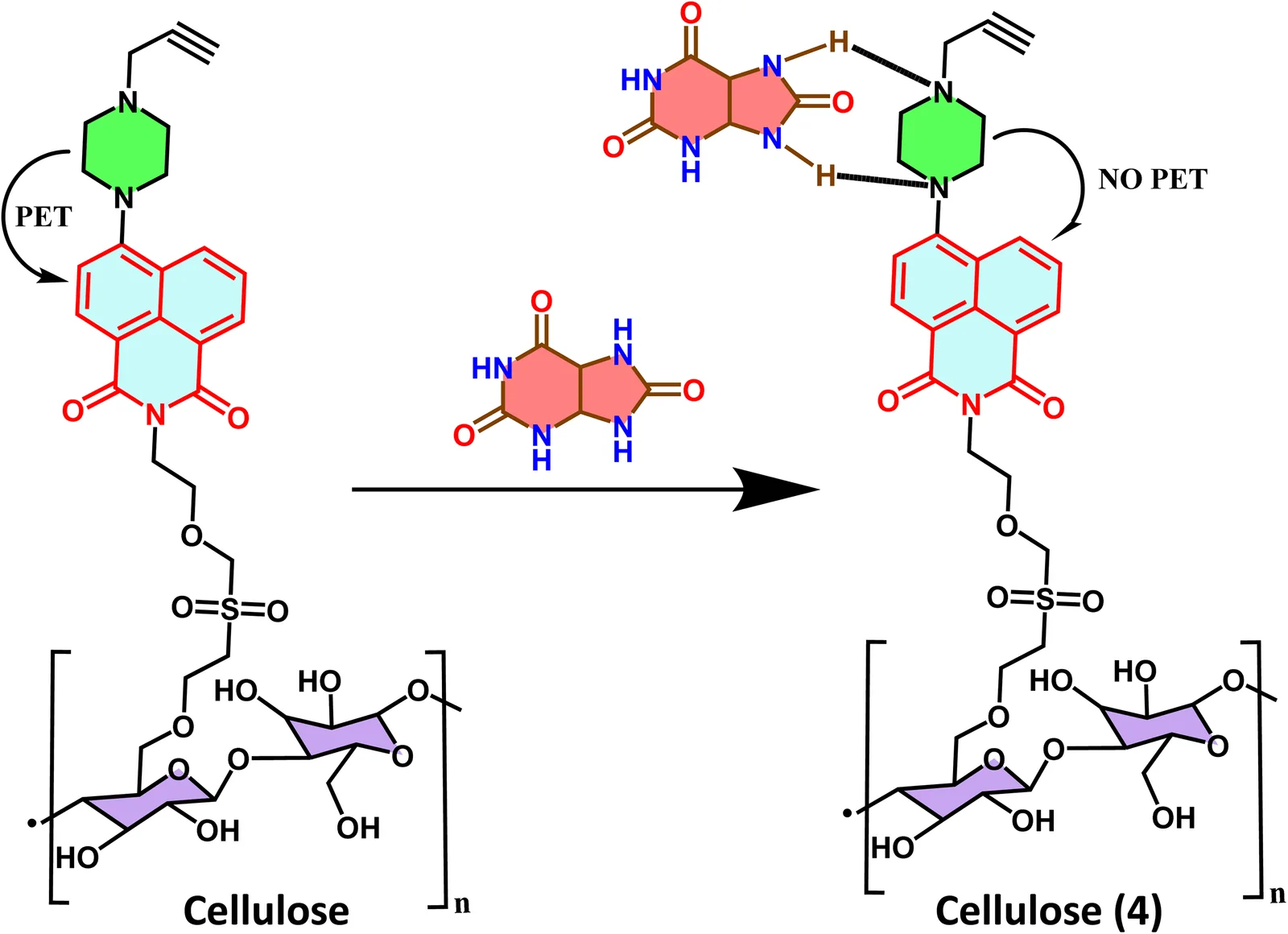
A schematic illustration of the mechanism by which chemosensor 4 recognizes UA.

Sidhu *et al.*, reported the modification of naphthalimide unit using piperazine ring, increased the Stokes shift, thereby enhancing the optical properties of the unit.^37^ The naphthalimide functions as a proton acceptor and forms a hydrogen bond with uric acid. Following coupling with allyl bromide, the piperazine-linked naphthalimide (5) (Fig. 6A) exhibited photoinduced electron transfer (PET) through tertiary ‘N’ of the piperazine ring, which suppresses the fluorescence. Upon interaction between uric acid and the lone pair on ‘N’ of piperazine, the fluorescence intensity increases at a wavelength of 540 nm. UV-vis studies indicated that a stable band at 410 nm of 5 remains unchanged after the addition of uric acid. Fluorescence titration studies with various biomolecules revealed that, uric acid, compared to other biomolecules, induced significant changes in intensity due to the participation of probe 5 as a receptor through H-bonding with uric acid (Fig. 6C). Furthermore, a gradual increase in uric acid concentration resulted in a linear enhancement of the emission intensity (Fig. 6B). Cellular imaging experiments in HeLa cells demonstrated strong green fluorescence was obtained in the presence of 5–20 µM concentration of uric acid with an incubation time of 2 hours, following treated with the probe 5 for 30 min. The cell viability assay determined that concentrations up to 10 µM of compound 5 were acceptable for biocompatibility in biomedical applications.

**Fig. 6:**
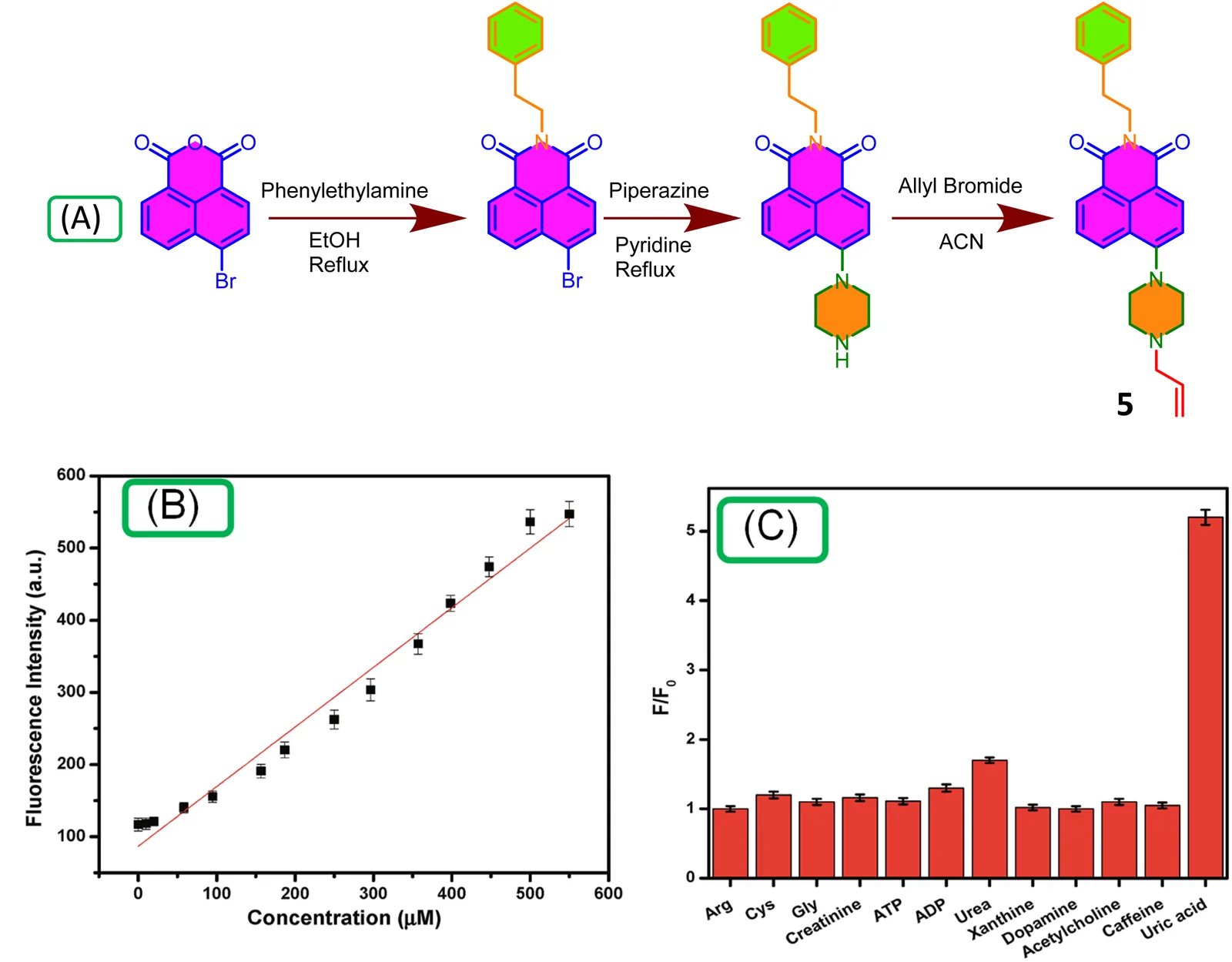
(A) The synthetic procedure of the sensor probe 5. (B) After the successive addition of uric acid to probe 5, a linear increase in the emission spectra. (C) Investigation of the probe's selectivity for uric acid and other relevant biomolecules (this figure has been adapted from ref. 37 with permission from Royal Society of Chemistry, copyright 2019).

Dey *et al.* developed a conventional host–guest system (6a–6c) for the detection of uric acid, incorporating a set of donor and acceptor atoms (Fig. 7). The designed and synthesised naphthyridine-based four water soluble receptors (6a–6c) exhibit superior performance in binding with uric acid in an aqueous medium. UA contains complementary hydrogen-bond donor (D) and acceptor (A) sites.

**Fig. 7:**
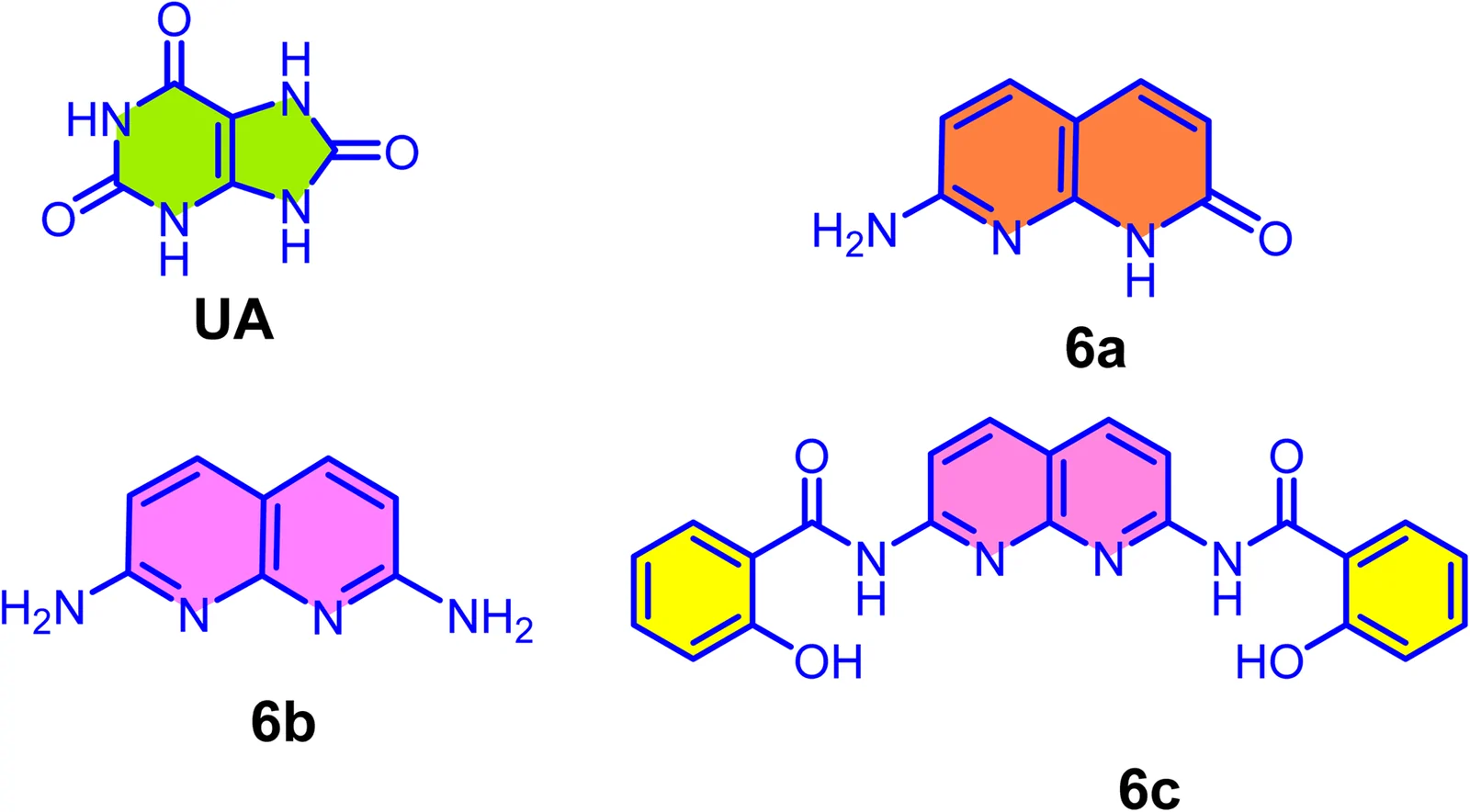
Structure of UA, 6a, 6b, and 6c probe.

Among the three systems, 6b exhibited higher binding activity to UA relative to 6a and 6c. This enhanced activity is attributed to the presence of the ADDA binding system in 6b, which is associated with uric acid.^38^ In the case of 6a, self-dimerization occurred over uric acid binding, reducing its binding affinity. The receptor 6c contains two H-bond acceptor systems (two N- atoms of the pyridine ring) and four H-bond donor systems (two –NH–CO and two –OH groups), which can efficiently bind with UA. However, due to conformational flexibility, 6c forms intra-molecular H-bonding in the solution (Fig. 7 and 8). Upon complex formation with 6b, the fluorescence intensity is quenched, indicating that 6b emerges as a promising candidate for high sensitivity towards UA and lower detection limit. This work advances the fabrication of water-soluble, non-enzymatic fluorescent probes for UA detection, which may have applications in medical scenarios.

**Fig. 8:**
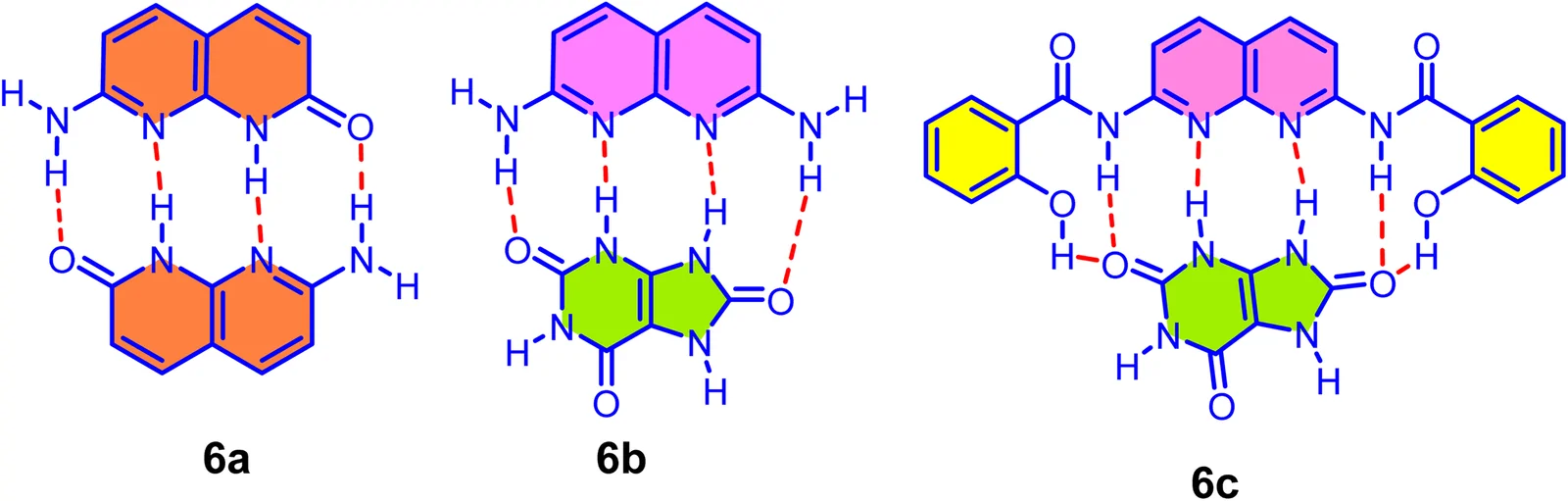
(1) Self-dimerization structure of probe 6a; (2) complex of receptor 6b receptor with UA; and (3) complex of receptor 6c receptor with UA using donor–acceptor H-bonding interactions.

#### 2.1.2 Chemosensors having metal ions as coordination sites (MOFs, coordination polymers)

Yan and colleagues developed a stable and luminescent phosphonate Ln-MOFs MIL-91(Al : Eu) and its derivatives composite, Cu^2+^@MIL-91(Al : Eu) for the selective identification of UA.^39^ The MIL-91(Al : Eu) was synthesized using a solvothermal method. The emission peaks observed in MIL-91(Al : Eu) MOFs at 579, 589, 612, 652, and 702 nm are attributed to the ^5^D_0_ → ^7^F_*J*_ (*J* = 0, 1, 2, 3, 4) transitions. Notably, the ^5^D_0_ → ^7^F_2_ transition at 612 nm exhibits the highest emission intensity. In the Cu^2+^@MIL-91(Al : Eu) composite (7; Fig. 9), the presence of Cu^2+^ resulted in the suppression of Eu^3+^ emission, with luminescence being restored in the presence of UA. Among various species in urine, the composite selectively recognizes UA, with a detection of 1.6 µmol L^−1^. Experimental results indicate that the composite sensor functions as an efficient turn-on luminescence sensor for UA. Furthermore, real urine samples were analyzed to detect abnormal UA concentrations, demonstrating the potential of composite 7 as a rapid diagnostic tool for hyperuricuria. The experimental findings suggest that the hyperuricuria can be assessed using the composite powders and test powers.

**Fig. 9:**
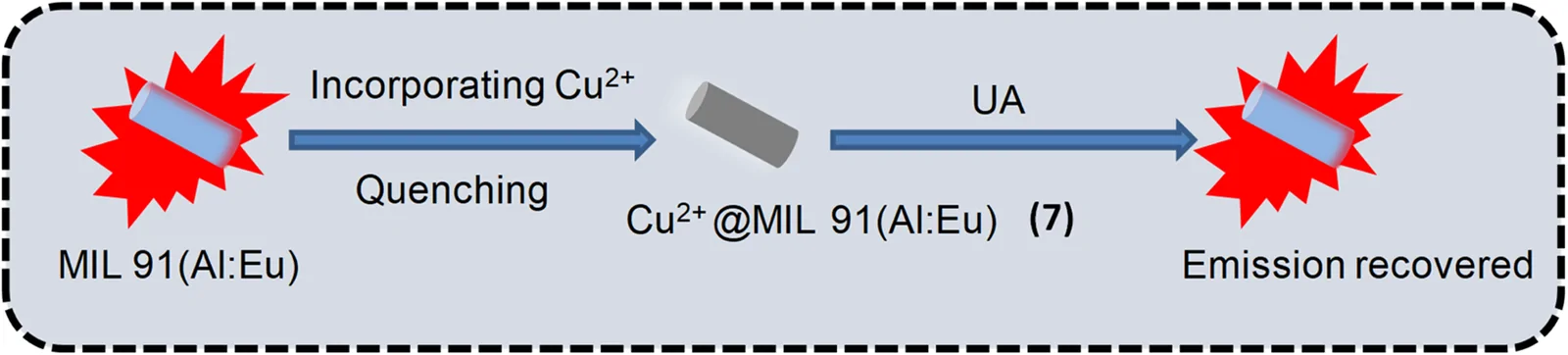
Schematic representation of the UA recognition mechanism by the sensor 7.

Gao and colleagues synthesized three novel isostructural fluorescent CPs, [L_*n*_(TCPB)(DMF)_3_]_*n*_, where L_*n*_ represents La (8), Ce (9), and Pr (10), with H_3_TCPB being 1,3,5-tris(1-(2-carboxyphenyl)-1*H*-pyrazol-3-yl)benzene (Fig. 10).^40^ All three polymers demonstrated ligand-centered fluorescence and exhibited substantial stability in aqueous media across a pH range of 3 to 10. UA induced fluorescence quenching in a concentration-dependent manner, with polymer 10 achieving the highest quenching efficiency at 84%, the largest Stern–Volmer constant of 7.91 × 10^3^ M^−1^, and the lowest detection limit of 0.28 µM. In comparison, polymer 8 showed a quenching efficiency of 81%, a Stern–Volmer constant of 6.68 × 10^3^ M^−1^, and a detection limit of 0.41 µM, while polymer 9 exhibited a quenching efficiency of 71%, a Stern–Volmer constant of 3.90 × 10^3^ M^−1^, and a detection limit of 1.12 µM. Additionally, polymer 10 demonstrated excellent selectivity, recyclability over six cycles, and satisfactory recovery rates ranging from 93.1% to 103.6% in synthetic urine.

**Fig. 10:**
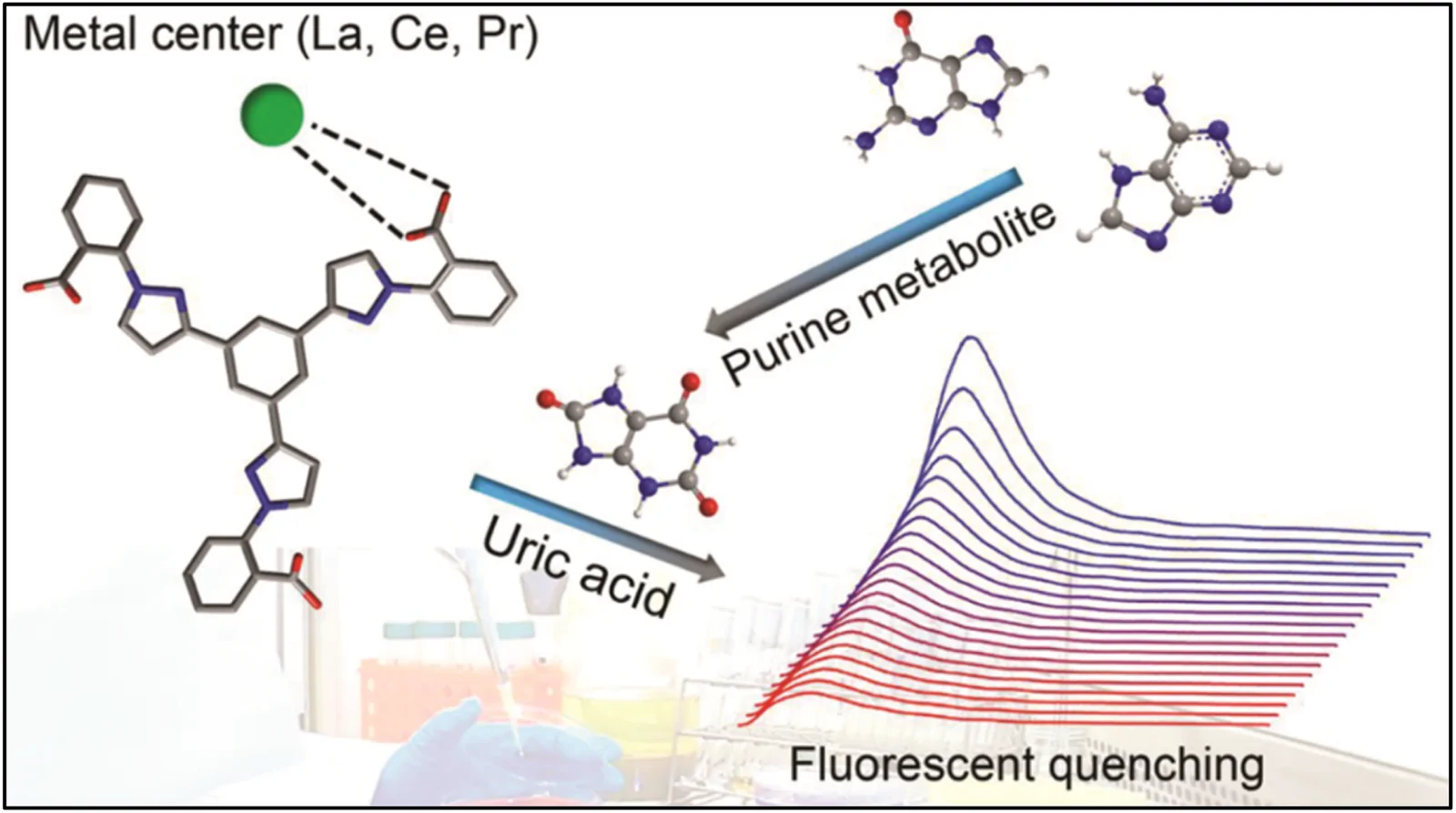
Schematic representation of the recognition strategy of UA by the coordination polymers 8–10 (where Ln = La, 8; Ce, 9 and Pr, 10) (this figure has been adapted from ref. 40 with permission from Royal Society of Chemistry, copyright 2020).

The authors primarily attributed quenching induced by UA to competitive absorption of excitation light, rather than to framework degradation or Förster Resonance Energy Transfer (FRET). PXRD confirmed that the coordination polymer (CP) structures remained intact post sensing. The absence of significant overlap between UA absorption and CP emission spectra was used to exclude FRET as a mechanism. Conversely, substantial overlap was observed between the excitation spectra of the CPs and UA absorption, with compound 10 showing particularly strong coincidence at 286 nm, which accounts for its superior response. However, the classifications of the quenching as static, based solely on linear Stern–Volmer plots, lacks mechanistic conclusiveness. The study did not include fluorescence-lifetime measurements, temperature-dependent Stern–Volmer analyses, or an independent quantitative correction for inner-filter effects. Consequently, it is not possible to unequivocally differentiate between static and dynamic quenching. The proposed mechanism of competitive excitation-light absorption aligns with an inner-filter-type attenuation mechanism; however, its contribution was not independently quantified. The evidence available supports competitive absorption as the primary proposed cause of fluorescence attenuation, while a definitive assignment of static quenching or exclusion of other excited-state pathways remains unsubstantiated. Notably, the superior performance of compound 10 is associated with its greater excitation/UA absorption overlap at 286 nm. The study, however, does not further elucidate how the different lanthanide centers alter the electronic environment responsible for this spectral variation. Additionally, the minimal spectral overlap between compound 10 and the tested interferents reduces competitive absorption and FRET involving these species, thereby enhancing to the high selectivity of compound 10 for UA in a mimetic physiological environment.

A luminescent chemosensor derived from a post-functionalized metal–organic framework, UiO-PSM (11; Fig. 11), was developed by Jia and colleagues for the direct recognition of UA.^41^ The UiO-PSM was synthesized through the amidation of the zirconium-based framework UiO-66-NH_2_ with 2-picolinic acid. The probe was specifically designed for direct UA sensing, as UA can suppress the fluorescence of the MOF through hydrogen bonding, coordination, and π–π interactions. Upon modification, UiO-PSM exhibited strong blue fluorescence at 435 nm when excited at 377 nm, with a quantum yield of 15.56%, indicating its suitability for luminescent sensing applications. The enhanced luminescence of UiO-PSM is attributed to energy transfer between the organic linker and the guest lanthanide ions. The metal organic framework (MOF) comprises Zr_6_ clusters, amide-linked picolinamide-functionalized 2-aminoterephthalate linkers, and Eu^3+^/Tb^3+^ ions coordinated to the free carboxylate groups.^42^ The organic linker functions as an antenna by absorbing light and reaching its excited triplet state (T_1_) through intersystem crossing. Subsequently, the excited linker transfers its energy to the lanthanide ions, which become excited and emit light. Therefore, efficient linker-to-lanthanide energy transfer is responsible for the enhanced luminescence of the MOF. The strong fluorescence of the UiO-PSM MOF is quenched upon addition of uric acid (UA). When UA enters the framework, it interacts with the functionalized linker and Zr sites through coordination, hydrogen bonding, and π–π interactions. These interactions form a relatively stable UiO-PSM–UA ground–state complex. This complex formation perturbs the electronic environment of the emissive linker and provides an additional non-radiative deactivation pathway, thereby reducing the population of the emissive excited state and resulting in fluorescence turn-off. UA-induced fluorescence quenching was investigated using UV–vis and FT-IR spectroscopy, Stern–Volmer analysis, and time-resolved fluorescence measurements. The results support a static quenching mechanism, characterized by a decrease in the Stern–Volmer constant (*K*_SV_) with increasing temperature, quenching rate constants (*K*_q_) values of approximately ∼10^12^ to 10^13^ L mol^−1^ s^−1^ exceeding the diffusion-controlled limit, and an essentially unchanged fluorescence lifetime (15.07 to 15.11 ns). Analytically, UiO-PSM enabled UA determination over a broad linear range of 0.01–400 µM, with a detection limit of 2.3 nM and a response time of approximately 1 min. The authors reported negligible interference from glycine, l-cysteine, adenine, guanine, glucose, urea, ascorbic acid, Na^+^, and K^+^. However, the selectivity assessment is incomplete because xanthine, hypoxanthine, glutathione, dopamine, ascorbic acid, and creatinine were not examined, and the concentrations of the tested interferents relative to UA were not clearly specified. Furthermore, mixed-analyte/competition experiments were not performed. This is particularly important for xanthine and hypoxanthine, which are closely related purine metabolites and structurally relevant analogues of UA. The probe's applicability was validated using serum and urine samples for UA detection. This novel research on UA recognition presents substantial potential for the clinical identification of disorders associated with UA.

**Fig. 11:**
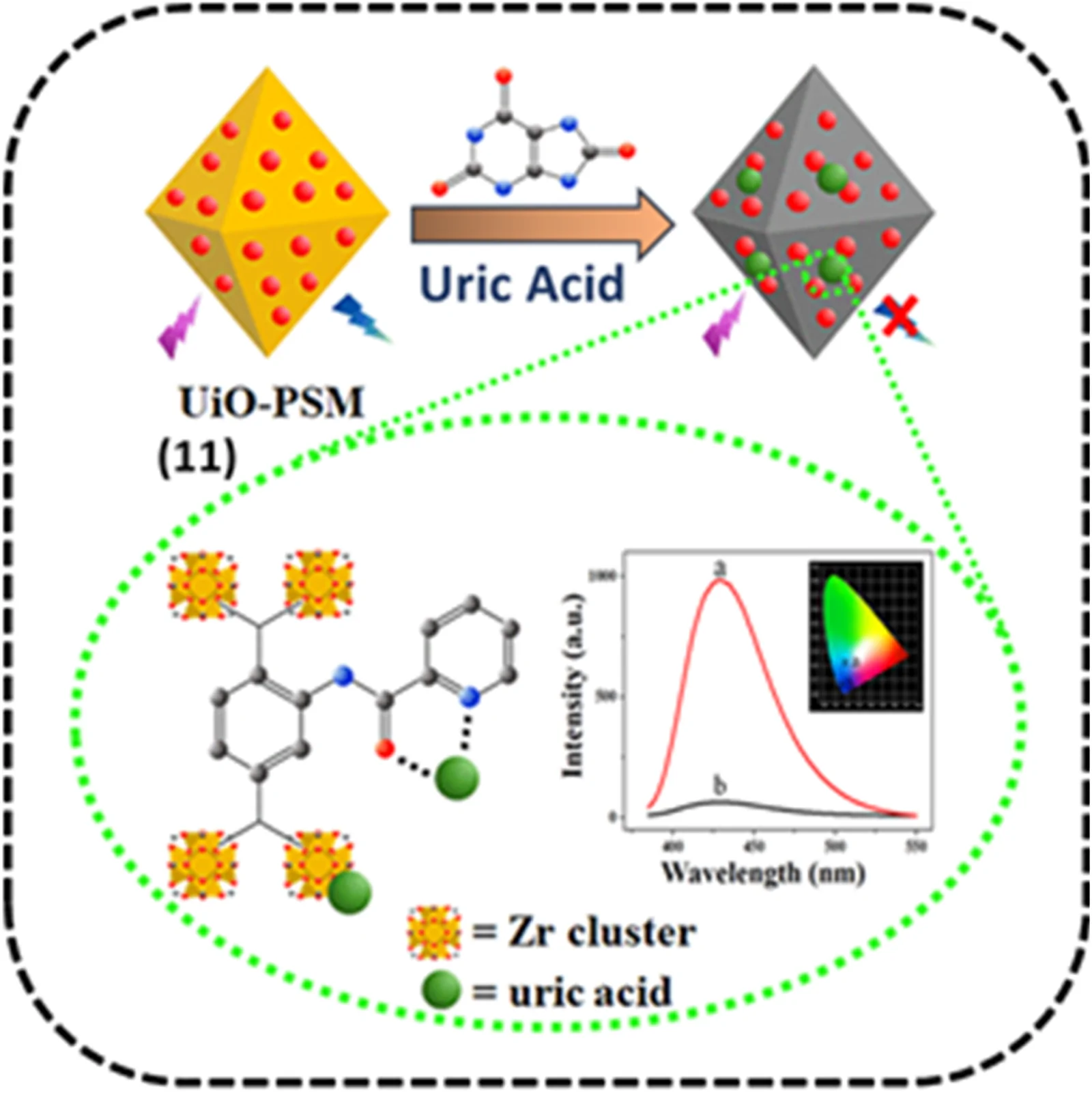
Schematic representation of the UA recognition mechanism by the MOF based sensor 11 (this figure has been adapted from ref. 41 with permission from American Chemical Society, copyright 2019).

Wang *et al.* developed a rapid and straightforward ratiometric fluorescent probe for ultrasensitive uric acid sensing through the successful synthesis of bovine serum albumin-protected bimetallic gold/silver nanoclusters (BSA-Au/Ag NCs) (12; Fig. 12), which exhibit significantly enhanced fluorescence in alkaline solution.^43^ The detection mechanism of this sensing platform, which employs a fluorescence quenching process, is predicated on the formation of 2,3-diaminophenazine (DAP) when *o*-phenylenediamine (OPD) is oxidised by H_2_O_2_. The generated DAP not only produces a distinct emission peak at 580 nm, but also effectively quenches the intrinsic fluorescence of BSA-Au/Ag NCs at 690 nm, thereby facilitating a reliable ratiometric signal with improved analytical precision and reduced environmental interference (Fig. 12). Importantly, this method ensures specificity and sensitivity in complex biological environments by using enzymatically generated H_2_O_2_ as a key step in UA detection. The ratiometric fluorescent sensing system demonstrated exceptional sensitivity, characterized by a linear range of 5.0 × 10^−6^ to 5.0 × 10^−5^ M and a low limit of detection of 5.1 × 10^−6^ M (S/N = 3). Its practical applicability was further corroborated by validation studies utilising blood samples, which showed high concordance with clinically validated procedures. This study presents a reliable, stable, and ultrasensitive sensing platform that is not only easily designed but also widely applicable for biomedical analysis, including H_2_O_2_-generating reactions, offering substantial potential for clinical diagnostics and bioanalytical applications.

**Fig. 12:**
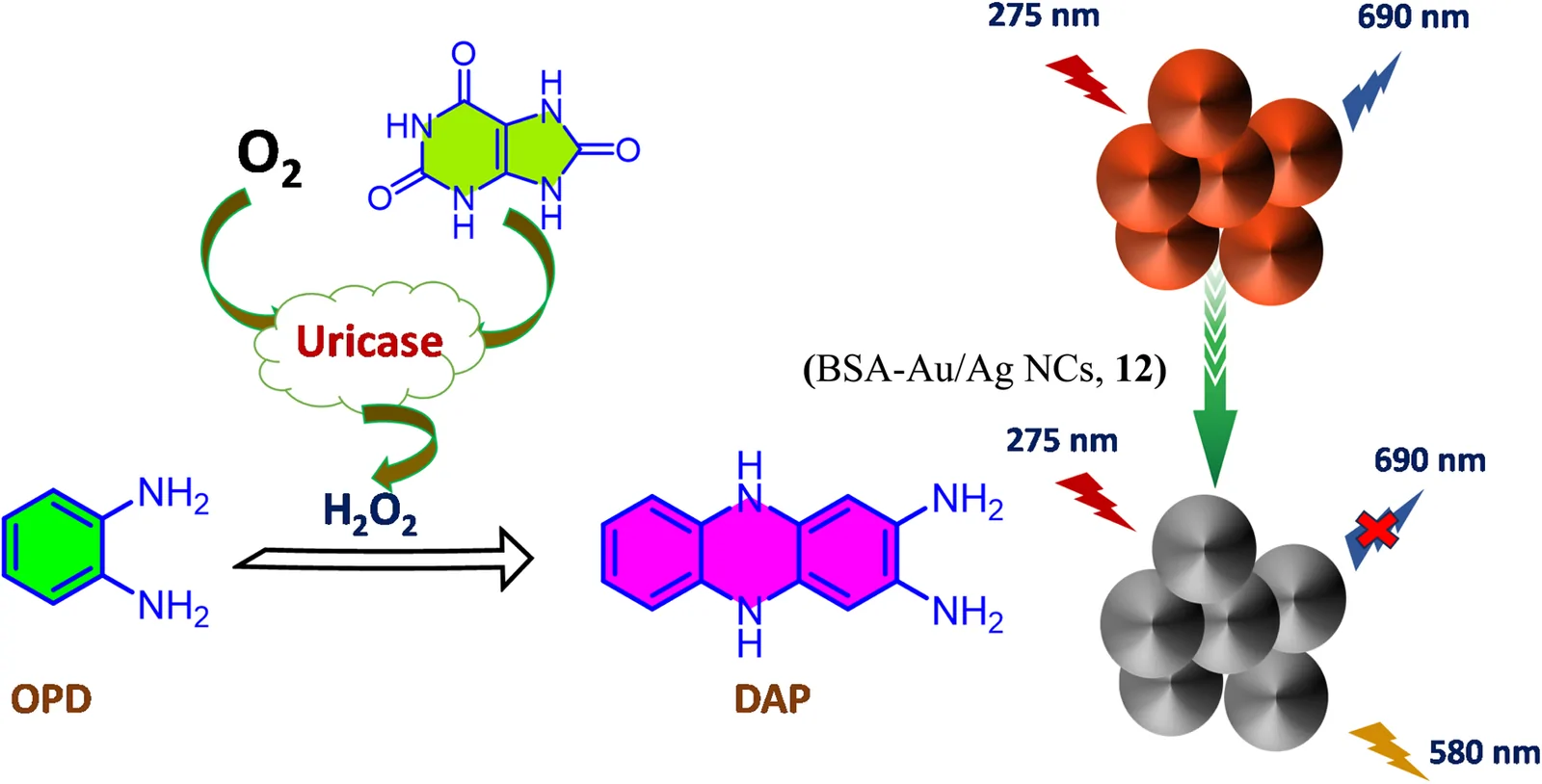
Schematic illustration of the measuring principle of the uric acid probe 12.

Jie and colleagues have an innovative composite material based on Cu^2+^ functionalized Tb-MOFs (13; Fig. 13) for the ultrasensitive detection of UA, a crucial metabolite of purine metabolism within biological systems.^44^ The synthesized Cu^2+^@Tb-MOFs demonstrate exceptional stability in water and across various pH levels, coupled with high selectivity and robust anti-interference capabilities, even amidst other components typically present in urine. The probe operates *via* a “turn-on” fluorescence mechanism, enabling reliable uric acid detection over a wide concentration range of 0–104 µM, with a detection limit as low as 0.65 µM. This advancement enhances the reliability of uric acid analysis and reduces the likelihood of false diagnosis. Furthermore, the integration of a molecular logic gate introduces an intelligent sensing strategy, underscoring the potential of this system not only as an efficient fluorescence sensor for uric acid in biological samples but also serving as a versatile platform for next-generation biochemical sensors and logic-based fluorescence devices (Fig. 13). Here, we represent a systematic comparison table of various sensing platforms used for UA detection with their medium, pH, matrix/sample, linear range, LOD, response time and dilution factor in Table 1.

**Fig. 13:**
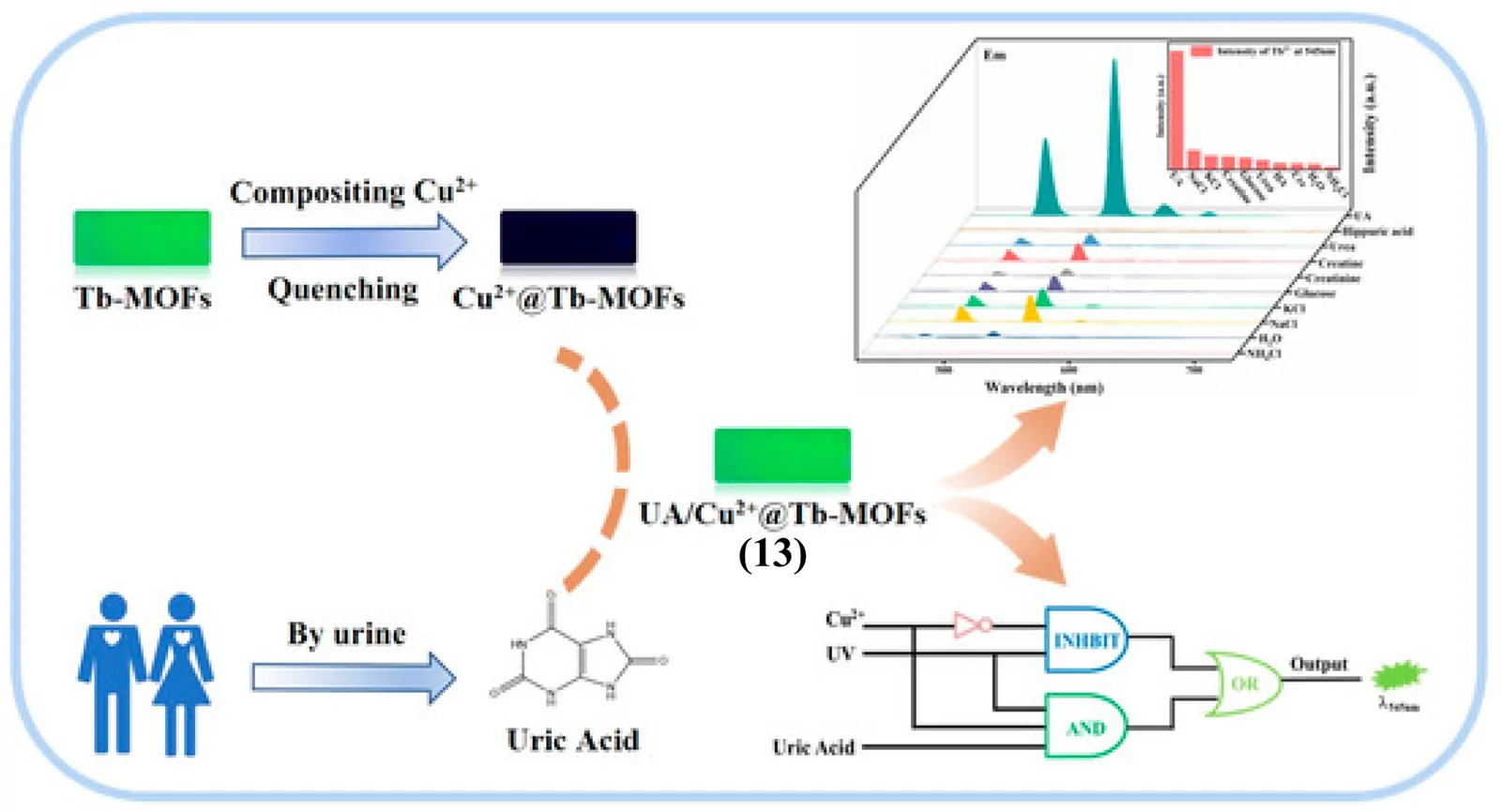
Schematic diagram of UA detection by the probe 13 and its logic gate application (this figure has been adapted from ref. 44 with permission from MDPI, copyright 2022).

**Table 1:** The summary table of sensor/platform, medium, pH, matrix/sample, linear range, LOD, response time and dilution factor

Sensor/platform	Medium	pH	Matrix/sample	Linear range	LOD	Response time	Dilution factor
Rhodamine–naphthyridine probe 1	Aqueous medium	NR	HeLa cells	NR	NR	NR	NR
Pyrene-appended thymine probe 2	10 mM HEPES buffer	7.4	Aqueous medium; live cells	NR	NR	NR	NR
Naphthyridine probe 3	H_2_O/DMSO (9 : 1), 10 mM HEPES	7.4	Urine, blood serum, Vero 76 cells	NR	0.6 µM	NR	NR
Piperazine-linked naphthalimide 4/S3/5	Aqueous medium	NR	HeLa cells	NR	NR	30 min probe incubation + 2 h UA incubation	NR
Cu^2+^@MIL-91(Al : Eu) 7	Aqueous medium	NR	Human urine	NR	1.6 µM	NR	NR
Ln coordination polymers 8–10	Aqueous medium	NR	NR	NR	0.41 µM (8); 1.12 µM (9); 0.28 µM (10)	NR	NR
UiO-PSM 11	Aqueous medium	NR	Serum, urine	0.01–400 µM	2.3 nM	1 min	NR
BSA-Au/Ag nanoclusters 12	Alkaline aqueous medium	NR	Blood	5–50 µM	5.1 µM	NR	NR
Cu^2+^@Tb-MOF 13	Aqueous medium	NR	Urine	0–104 µM	0.65 µM	NR	NR
N,P-CDs/Au@AgNPs 14	Aqueous medium	NR	Human urine	0.1–10 µM (colorimetric); 0.5–10 µM (fluorometric)	40 µM (colorimetric); 400 µM (fluorometric)	NR	NR
PVP–CuNCs/uricase 15	Aqueous medium	NR	Human urine	0.5–10 µM	113 nM	NR	NR
N–C dot/Cr(vi) 16	Aqueous medium	NR	Experimental samples	NR	12.5 nM	NR	NR
Zn-CD@Eu/DPA 17	Aqueous medium	NR	Natural water; blood serum; sweat	0–100 µM	0.36 µM	NR	NR
ssDNA-SWCNT CoPhMoRe 18	Aqueous/urine medium	pH-dependent	Urine	5.7–500 µM	NR	Fast (exact time NR)	NR
TMB–HAuCl_4_20	Aqueous medium	Optimized pH; exact value NR	Human urine	0.001–8.0 µM and 8.0–80.0 µM	0.67 nM	Optimized incubation; exact time NR	NR
MoS_2_/TMB 21	Aqueous medium	Optimized; exact value NR	Biological samples	0.5–100 µM	0.3 µM	NR	NR
Uricase/Cu^2+^/TMB 22	Aqueous medium	NR	Human urine	1–100 µM	0.64 µM	NR	NR
Ni@MnO_2_/TMB 23	Aqueous medium	NR	Human serum	1–40 µM	0.24 µM	NR	NR
IL-capped NiNPs 24	Aqueous medium	NR	Human serum	0.01–2.4 µM	0.13 µM	NR	NR
IL-capped NiNPs/TMB 25	Aqueous medium	7.5	Human serum	0.01–2.4 µM	0.13 µM	4 min	NR
Fe_4_Ni nanoflowers 26	Aqueous medium	NR	Serum	2–500 µM	1.13 µM	Fast; exact time NR	NR
AgNPs@MCA 27	Aqueous medium	NR	Human urine	20–100 µM	1 µM	NR	NR
Ag nanoprisms 28	Aqueous medium	NR	Blood	10–3000 nM	10 nM	NR	NR
Uricase–Ag nanoprisms 29	Aqueous medium	NR	Human serum	1–40 µM	0.7 µM	∼1 h	NR
N-CQDs/AgTNPs 30	Aqueous medium	NR	Human urine	0.1–45 µM	NR		

#### 2.1.3 Nanoparticle based fluorescent chemosensors for UA detection

Amjadi and co-workers synthesized nitrogen and phosphorus co-doped carbon dots (N,P-CDs) that emit green light (14; Fig. 14).^45^ Utilizing these N,P-CDs, they developed dual-mode sensing system with colorimetric and fluorometric outputs for the detection of uric acid. Such remarkable optical signal was attributed to the *in situ* formation of a silver shell around gold nanoparticles (AuNPs). Due to the spectral overlap between the emission of the carbon dots and the surface plasmon resonance (SPR) band of the AuNPs, the fluorescence of the N, P-CDs was significantly quenched. The SPR intensity of the AuNPs increased upon introduction of Ag^+^ ions and uric acid, as the Ag^+^ ions were reduced to metallic silver, forming a silver shell on the AuNPs. As the concentration of uric acid increased, the SPR peak shifted toward shorter wavelengths and the solution color gradually changed from red to orange. These shifts in SPR intensity and wavelength further enhanced the luminescence quenching of the N,P-CDs, due to greater spectral overlap with the resulting Au@AgNPs. The calibration ranges reported for the colorimetric and fluorometric modes were 0.1–10 µM and 0.5–10 µM, respectively, with applicability to human urine. Based on, these findings, a dual-mode sensor was successfully developed for uric acid detection, achieving detection limits of 40 nM using the colorimetric approach and 400 nM with the fluorometric method. The sensor demonstrated effective applicability in determining uric acid levels in human urine samples.

**Fig. 14:**
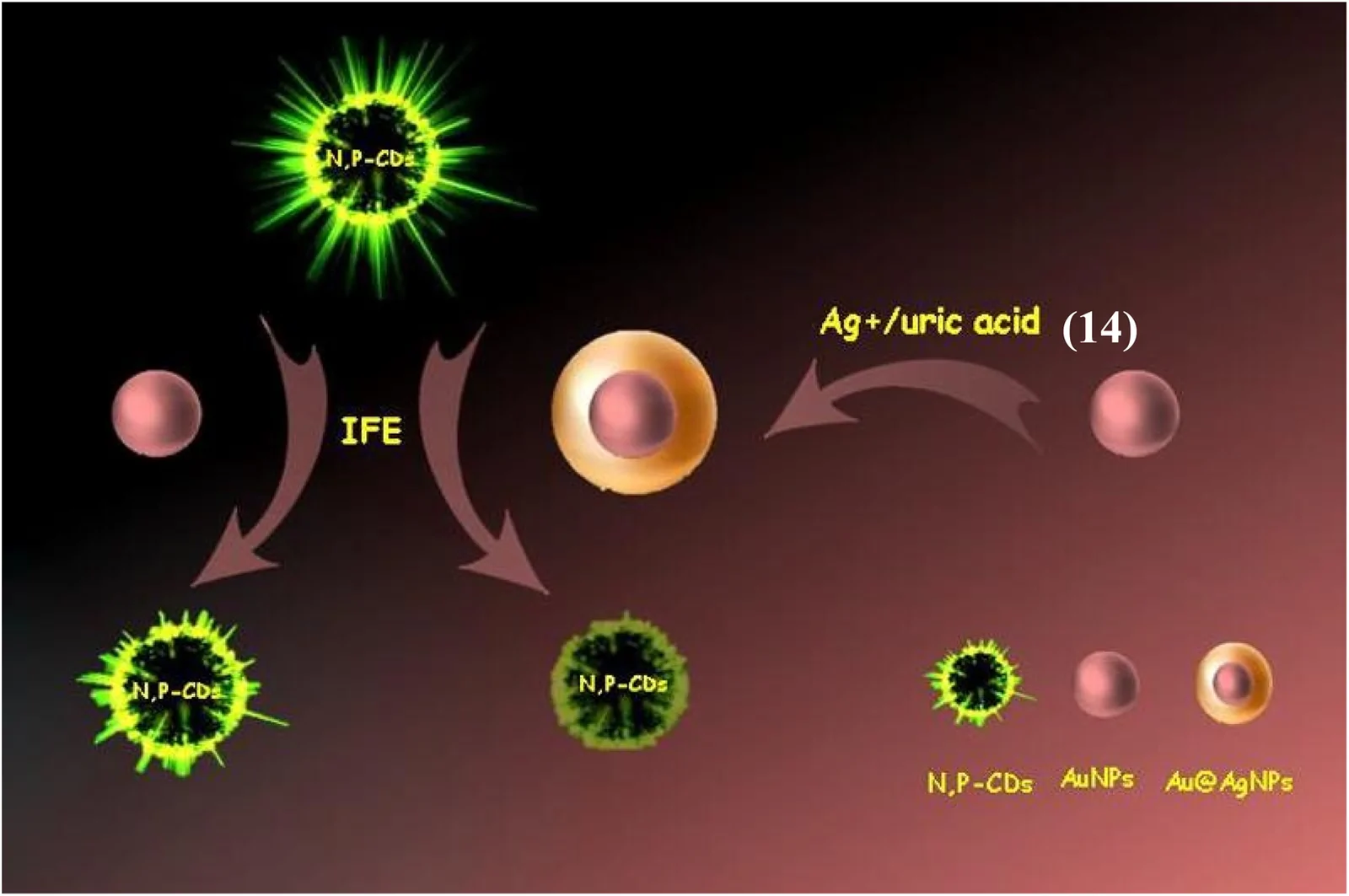
Schematic representation of the mechanism of UA recognition by the chemosensor 14 (this figure has been adapted from ref. 45 with permission from Elsevier, copyright 2021).

Ju and colleagues synthesized a poly-(vinylpyrrolidone) templated copper nanoclusters (PVP–CuNCs) (15; Fig. 15) for the detection of uric acid (UA).^13^ The PVP–CuNCs were produced through a straightforward wet chemical method, and their morphology and photophysical properties were characterized using high-resolution transmission electron microscopy and optical spectroscopic techniques. Upon excitation at 382 nm, the PVP–CuNCs showed strong emission at 430 nm. The researchers introduced a novel method utilizing uricase for the quantification of uric acid (UA) in aqueous environments including human urine. Polyvinylpyrrolidone-copper nanoclusters (PVP–CuNCs) functioned as luminescent indicators in the enzyme-catalyzed oxidation of UA by uricase. Upon the introduction of UA into the PVP–CuNCs/uricase solution, the oxidation reaction ensued, yielding H_2_O_2_, allantoin, and carbon dioxide. The generation of H_2_O_2_ in the reaction medium resulted in the quenching of 430 nm emission of PVP–CuNCs, with quenching efficiency increasing proportionally to UA concentration. A linear relationship with a negative slope was observed between fluorescence intensity and UA concentration within the range of 5–100 × 10^−7^ mol L^−1^. The limit of detection for UA was estimated to be 113 × 10^−9^ mol L^−1^. Furthermore, the luminescent nanocluster probe demonstrated strong selectivity for UA over competing biological molecules. This high sensitivity and selectivity highlight the potential of PVP–CuNCs for sensitive and cost-effective UA monitoring in human urine for clinical diagnostic applications.

**Fig. 15:**
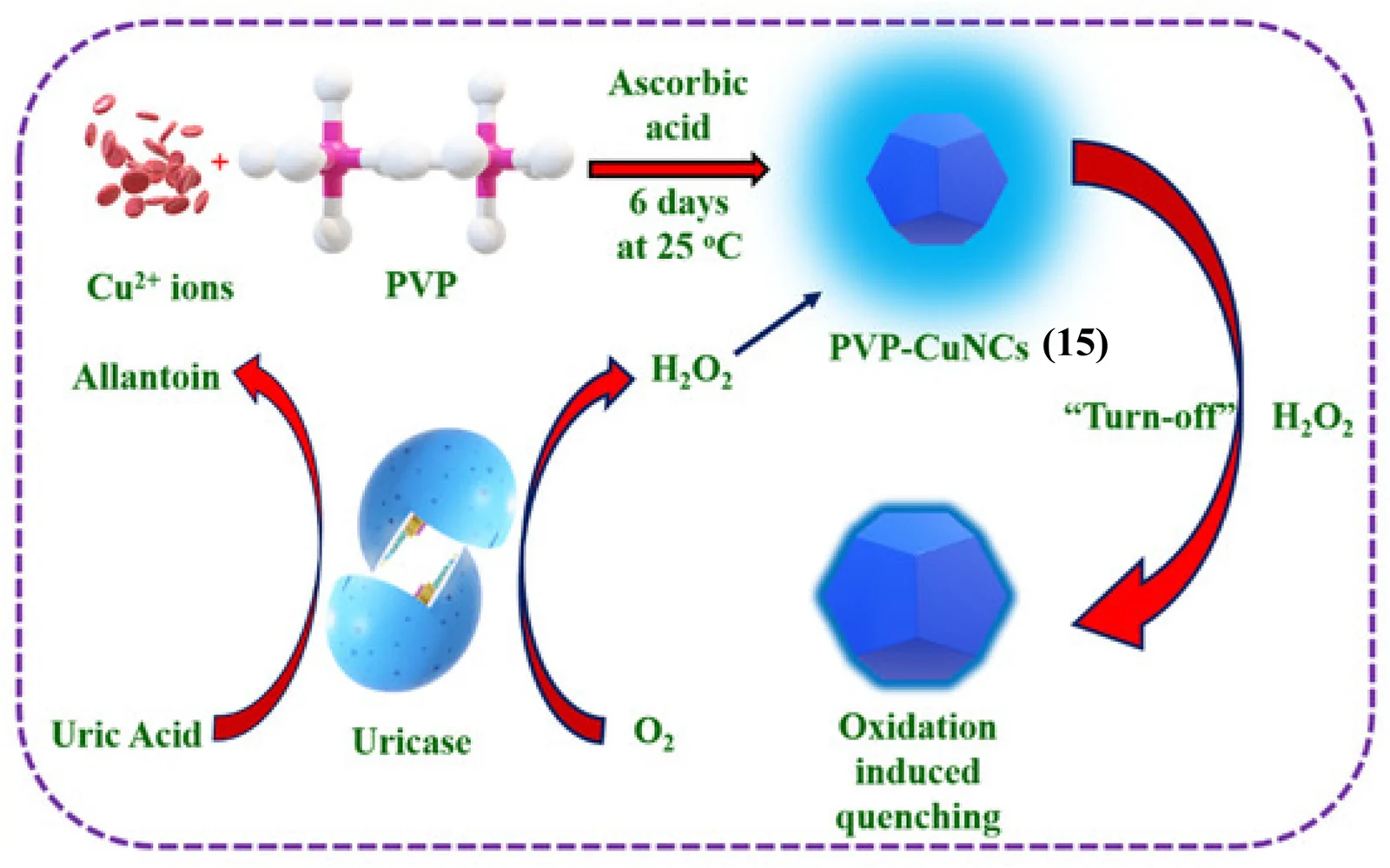
Schematic for oxidation of UA with uricase, followed by the H_2_O_2_-induced emission quenching of PVP–CuNCs (15) (this figure has been adapted from ref. 13 with permission from MDPI, copyright 2023).

Vergheese and colleagues reported the synthesis of a luminescent nitrogen-doped carbon dot/chromium(vi) [N–C dot/Cr(vi)] (16; Fig. 16) nanoprobe *via* a green synthesis route for nanomolar detection of UA.^46^ The N–C dot/Cr(vi)-based nanoprobe was developed through a rapid, straight forward, solvent-free, and eco-friendly pyrolysis method. Various characterization techniques, including UV–vis, FTIR, XRD, SEM-EDX, and PL spectroscopy, were utilized to analyse the material. The nitrogen-doped carbon dots (N-CDs) exhibited strong fluorescence centred at approximately 475 nm when excited at 350 nm. The introduction of Cr(vi) into the N-CDs dispersion resulted in significant luminescence quenching, resulting in the forming the N–C dot/Cr(vi) nanocomposite. The addition of uric acid (UA) to this nanocomposite restored the blue luminescence by reducing Cr(vi) to Cr(iii)/Cr (0) through UA. This system achieved a detection limit of 12.5 × 10^−9^ M for UA. The findings indicate that the N-dot-based nanoprobe could be effective for detecting UA at nanomolar concentrations in experimental samples, with potential applications in real-world UA detection in the future.

**Fig. 16:**
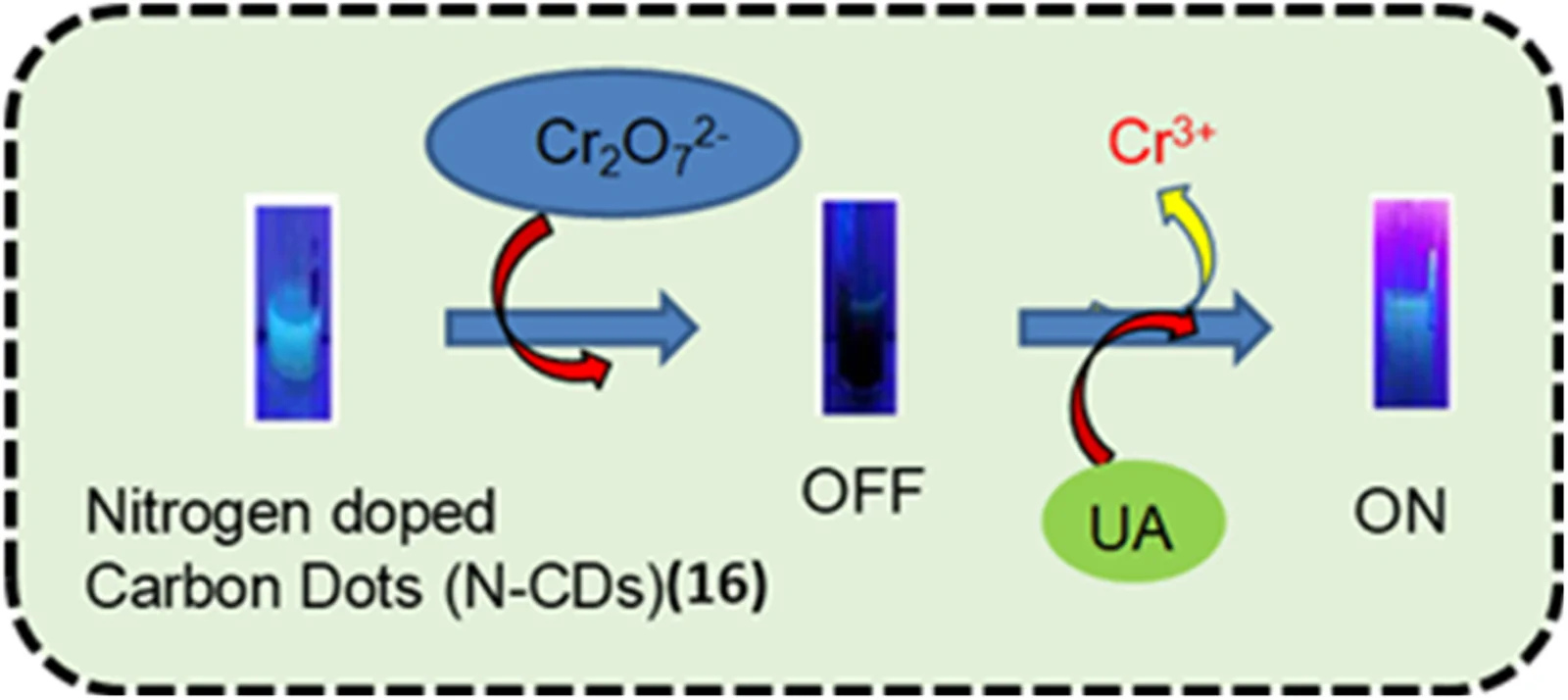
Schematic representation of the recognition of UA by the nanocomposite system 16 (this figure has been adapted from ref. 46 with permission from Springer Nature, copyright 2021).

A Zn-CD@Eu nanoprobe for the ratiometric detection of dipicolinic acid (DPA) and uric acid (UA) at ambient temperature was successfully synthesized by Mohapatro and colleagues.^47^ Upon interaction with DPA, the nanoprobe forms Zn-CD@Eu/DPA (17; Fig. 17), which subsequently reacts with UA, transforming into a dual-emissive platform. Upon excitation at 360 nm, Zn-CDs exhibit blue emission at 437 nm. The emitted photon from Zn-CDs is absorbed by the dipicolinic acid (DPA) moiety, which becomes excited to the triplet state. DPA functions as an antenna ligand, absorbing the excitation energy and transferring it to Eu^3+^, thereby populating its excited state and facilitating the ligand-to-metal charge-transfer (LMCT) process.^48–50^ The excited Eu^3+^ subsequently undergoes the characteristic ^5^D_0_ → ^7^F_2_ transition, resulting the red emission at 616 nm. This process, generates a the ratiometric emission response in the sensing system. Consequently, an increase in UA concentration leads to a decrease in F_616_ along with a relative enhancement of the blue-to-red fluorescence ratio (F_437_/F_616_), producing the ratiometric analytical response.^51,52^ Uric acid (UA) disrupts this sensitized coordination environment, thereby suppressing the DPA-mediated energy-transfer pathway to Eu^3+^ and reducing the red Eu^3+^ emission. In contrast, the Zn-CD emission at approximately 437 nm remains largely unaffected. This dual-channel configuration offers advantages over single-wavelength fluorescence measurements, as the intensity ratio minimizes errors arising from probe concentration, excitation fluctuations, optical path length, and instrumental variations. The Zn-CD@Eu/DPA platform thus provides a quantitative UA response over 0–100 µM with a reported detection limit of 0.36 µM. Researchers evaluated the probe's performance in real samples by testing it with spiked solutions of blood serum, sweat, and natural water sources. This non-enzymatic approach circumvents the common limitations of traditional enzyme-based uric acid sensors, such as high costs and the susceptibility of proteins to denaturation. Furthermore, the nanoprobe demonstrates excellent water solubility, biocompatibility, and stability, achieving high recovery rates in both environmental and biological samples. In addition to sensing, the platform facilitates *ex vivo* fluorescence imaging to visualize uric acid accumulation within the Malpighian tubules of *Drosophila melanogaster*. Its selectivity, robustness, and dual capability for detection and imaging underscore its significant potential for clinical diagnostics, environmental monitoring, and multidisciplinary research applications.

**Fig. 17:**
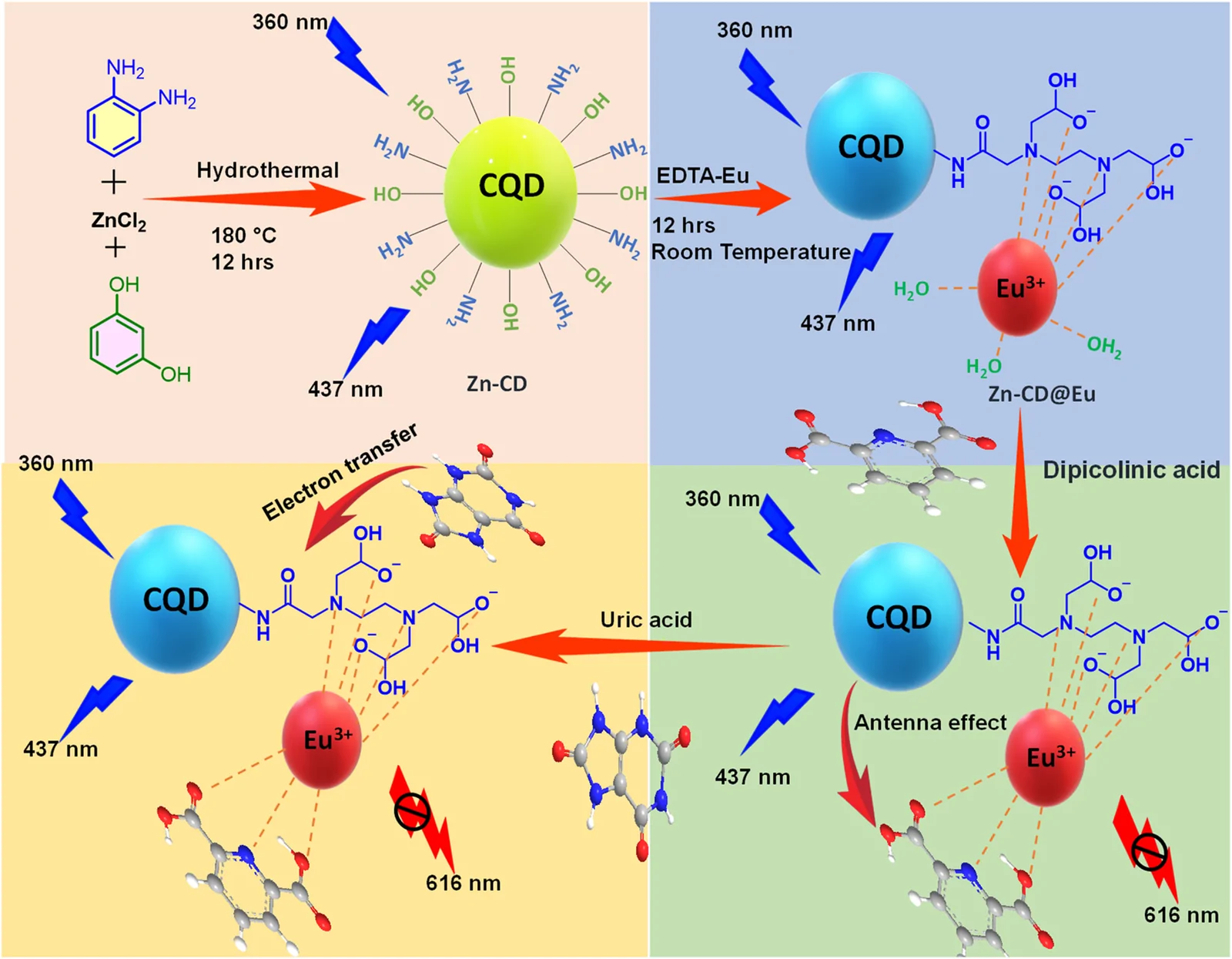
Schematic illustration of the synthesis of Zn-CD@Eu and sensing of DPA and UA (17).

In addition to chemosensors derived from small molecules, MOFs, coordination polymers, and nanoparticles, Cho and colleagues developed single-stranded DNA (ssDNA) functionalized single-walled carbon nanotubes (SWCNTs) to create a new fluorescent chemosensensor for uric acid. Utilizing the corona phase molecular recognition (CoPhMoRe) strategy, they engineered a NIR optical nanosensor for enzyme-free uric acid detection (18; Fig. 18).^53^ The study identified that (AAT)\_10_/SWCNT provided the most effective corona phase, which was further optimized through pH-dependent analysis to accommodate variations in urinary pH. This corona nanosensor was integrated with an optical paper strip for point-of-care (POC) applications. When tested with urine samples containing 5.7–500 µM UA, the nanosensor generated a exhibited a rapid and significant turn-on NIR signal, increasing nearly fourfold. The corona nanosensor-based paper strip also demonstrated stable and reliable UA detection under challenging conditions (40 °C and 70% relative humidity), maintaining functionality for up to 15 days (Fig. 18). The work underscores the applicability of SWCNT-based CoPhMoRe as an optical point-of-care testing platform capable of enzyme-free UA detection in complex biofluid environments.

**Fig. 18:**
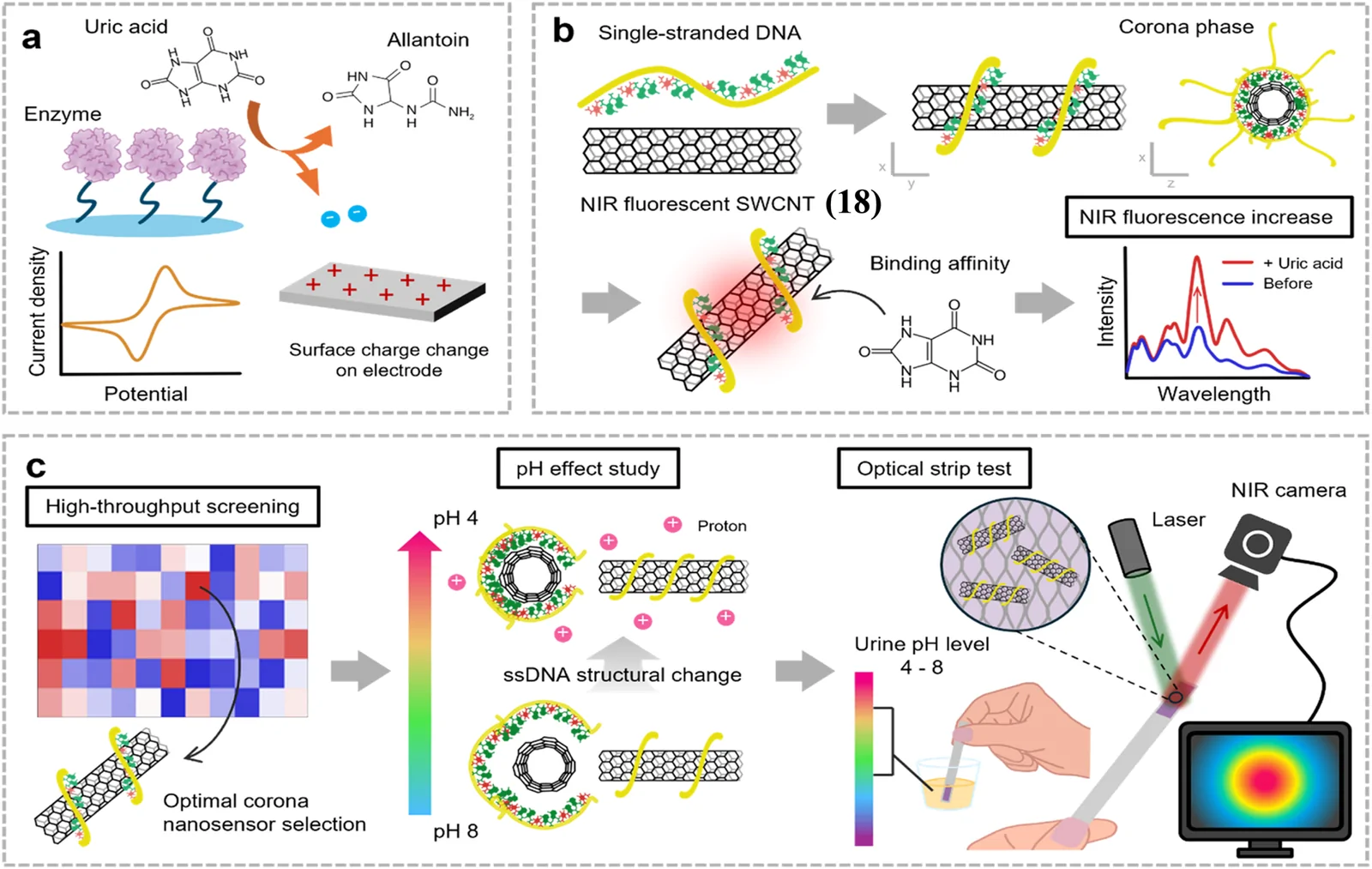
Illustration of enzyme-free uric acid monitoring employing CoPhMoRe-based NIR fluorescent SWCNTs (18). (a) Conventional enzymatic approach for UA detection *via* conversion to allantoin. (b) Development of ssDNA-functionalized SWCNT nanosensors for enzyme-free UA sensing using CoPhMoRe. (c) Schematic workflow for optimizing the corona nanosensor-based optical strip for urinary UA detection under varying pH conditions (this figure has been adapted from ref. 53 with permission from Royal Society of Chemistry, copyright 2025).

### 2.2 Colorimetric sensors

Colorimetric sensing platforms have gained substantial attention due to their simplicity, cost-effectiveness, rapid response, high sensitivity, selectivity, and the distinct color changes observable to the naked eye. Their portability ability to operate without instruments make them appealing for detecting a diverse array of analytes. The majority of UA colorimetric sensing systems are based on the following mechanisms.

(i) Uric acid induces the reduction of metal ions such as the conversion of Au^3+^ to Au^0^, resulting in a color change in the HAuCl_4_–dye system (Fig. 19A).

**Fig. 19:**
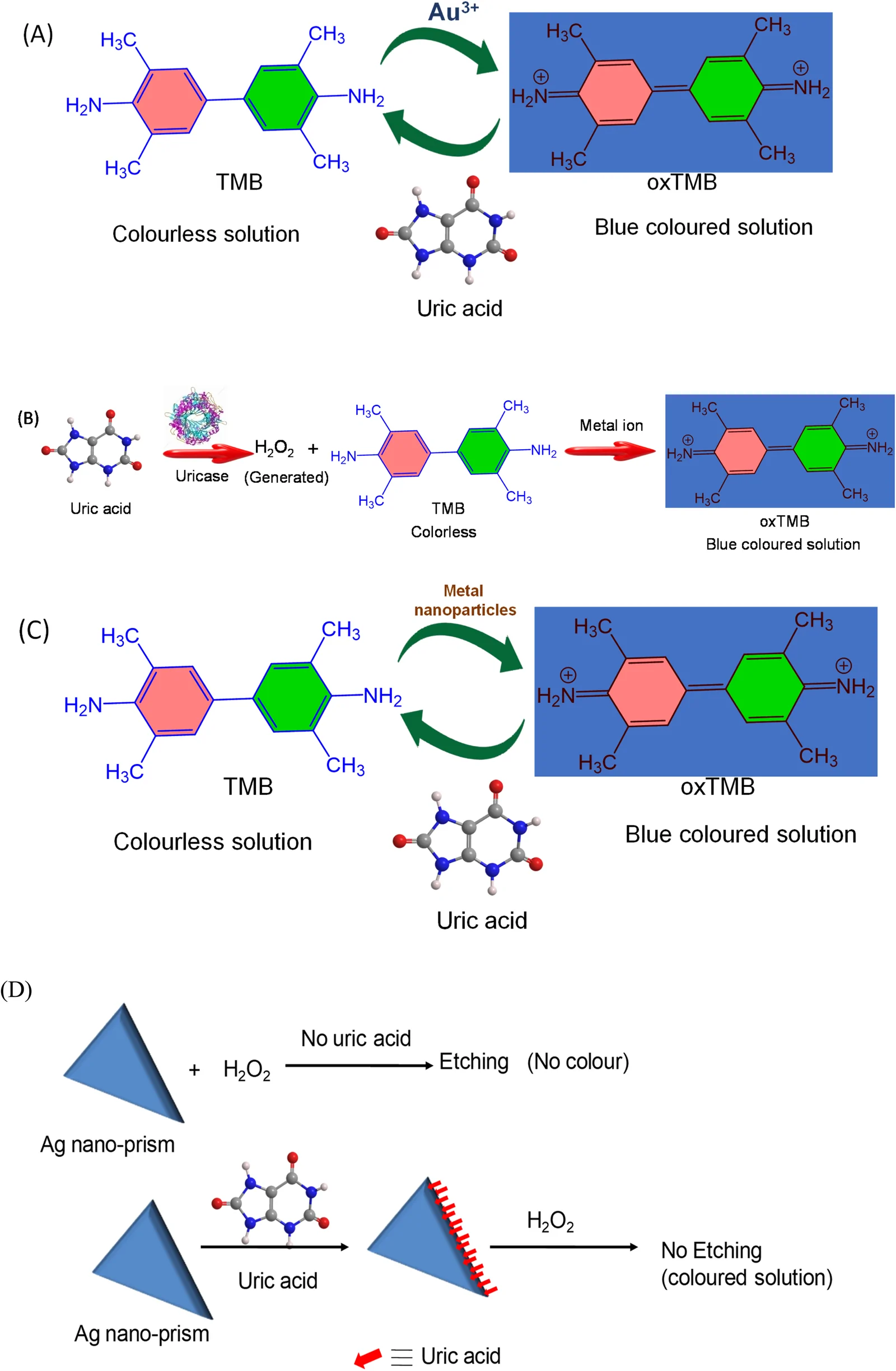
(A) Schematic illustration a Au^3+^-TMB based colorimetric sensor, Au^3+^ oxidized the TMB dye, which further reduced by UA and the blue color of the solution becomes colorless. (B) Schematic illustration a nanozyme based colorimetric sensor for UA. (C) Schematic illustration of UA recognition by metal nano-particle based colorimetric sensor. Metal nano-particle oxidized the dye in presence of H_2_O_2_ the solution becomes colored. In presence of UA the oxidized dye reverts back to the original form and solution becomes colorless. (D) Schematic representation of a nanoprism based colorimetric sensor.

(ii) Reaction with chromogenic substrates in presence of nanozymes (Fig. 19B).

(iii) Metal nanoparticles and hydrogen peroxide oxidize the dye to produce colored solution. The oxidized dye is subsequently reduced in the presence of UA, leading to a fading of the solution's color (Fig. 19C).

(iv) A facet-dependent etching mechanism employs unmodified silver nanoprisms, in which the (110) crystal face is attacked by H_2_O_2_ in the absence of UA, resulting in loss of solution color (Fig. 19D).

#### 2.2.1 TMB–HAuCl_4_ based colorimetric sensor system

Li group has developed a straightforward colorimetric sensor for detecting uric acid (UA) using 3,3′,5,5′-tetramethylbenzidine (TMB) in conjugation with HAuCl_4_ (20; Fig. 20). The reaction between TMB and varying concentration of HAuCl_4_ results in a color change from red to blue.^54^ The colorless TMB can be oxidized by HAuCl_4_ to form oxidized TMB (oxTMB), which exhibits a blue color with an absorbance maximum at 652 nm. In the presence of UA, the Au^3+^ ions of HAuCl_4_–TMB system reduced to Au^0^, leading to a reduction in the blue a decrease in absorption intensity at 652 nm. The group proposed that for the recognition of UA does not require any nanomaterials or organic probes. They also optimized the sensing conditions, including pH value, incubation time and the sensor system concentration. A linear sensing response was observed within the concentration range of 0.001–8.0 µM and 8.0–80.0 µM, with a detection limit of 0.67 nM (S/N = 3). This sensor is also capable of detecting uric acid in urine samples of human.

**Fig. 20:**
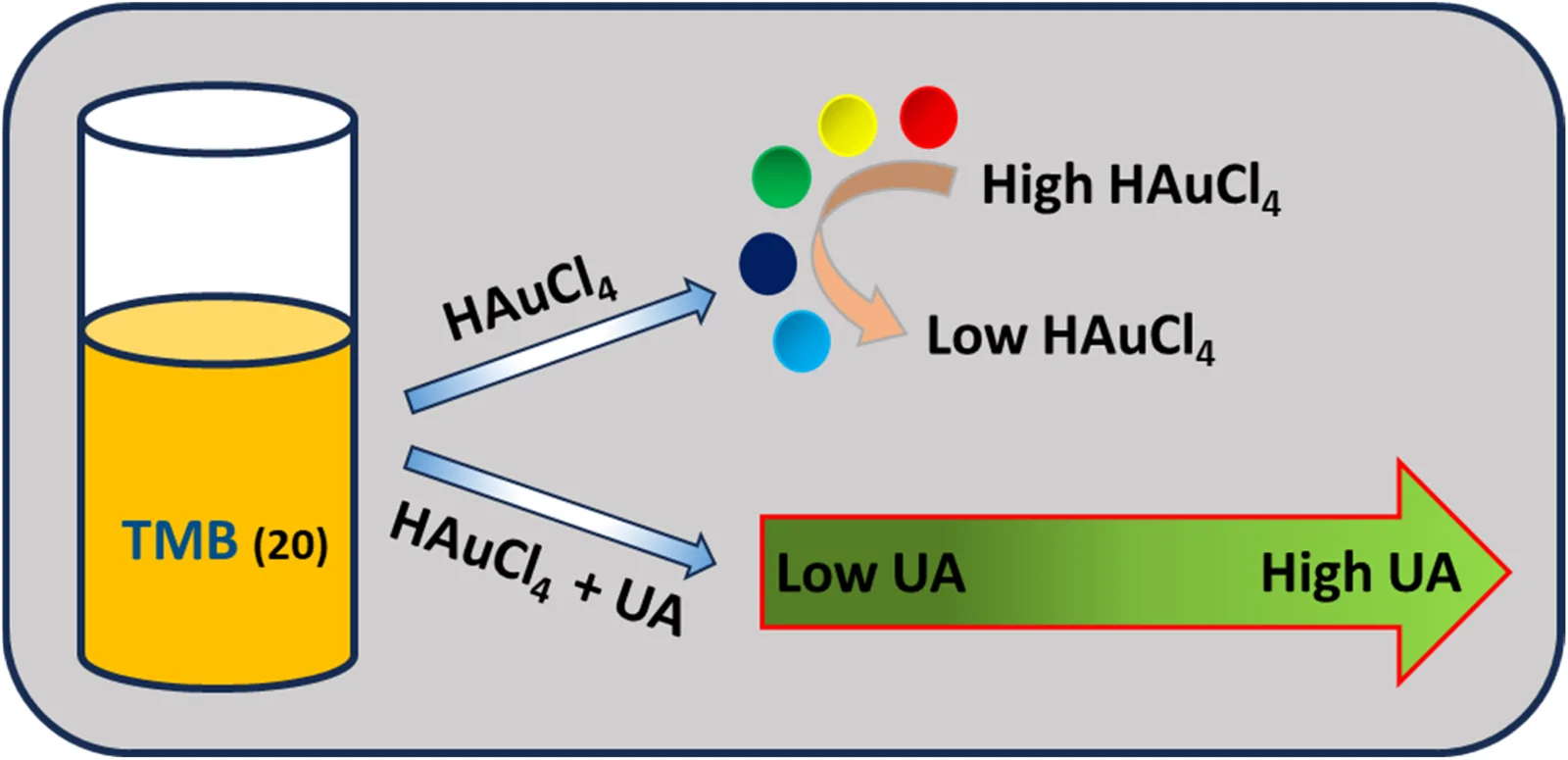
Schematic representation of the mechanism of UA recognition by the chemosensor 20.

Xue and colleagues introduced a straightforward and selective colorimetric assay for uric acid (UA) detection, utilizing MoS_2_-mediated oxidation of TMB (21; Fig. 21).^55^ The MoS_2_ nanoflakes in this system function as peroxidase-like catalysts, offering several advantages over natural enzymes, such as cost-effectiveness, high solubility in water, and superior catalytic stability. The assay demonstrated a strong linear dependence (*R*^2^ = 0.996) between the absorbance changes at 652 nm and UA concentration within the range of 0.5–100 µM under optimized conditions, achieving a detection limit of 0.3 µM (S/N = 3). Importantly, this sensing system does not require expensive analytical equipment, rendering it suitable for rapid point-of-care diagnostics. Here, we represent a systematic comparison table of different sensing platforms for UA detection in Table 2.

**Fig. 21:**
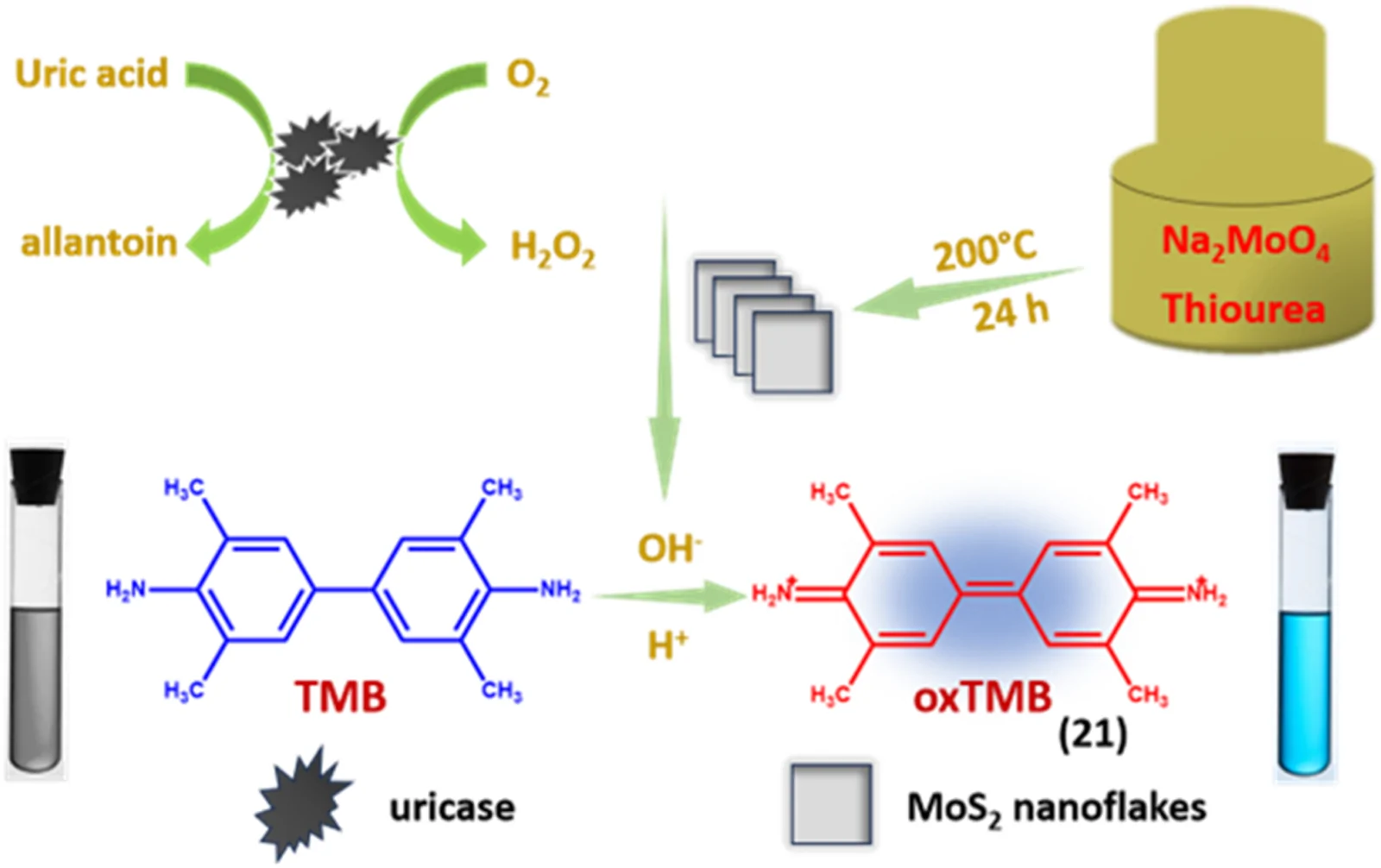
Schematic diagram of the colorimetric detection of UA using H_2_O_2_ and MoS_2_ catalytic oxidation of TMB (21).

**Table 2:** The summary table of sensing platforms, recovery/agreement with reference method, sample preparation, robustness (pH; temperature), stability, reproducibility and references

No.	Sensing platform	Recovery/agreement with reference method	Sample preparation	Robustness (pH; temperature)	Stability and reproducibility	Reference
1	Rhodamine–naphthyridine fluorescent turn-on probe (R1)	Serum recovery: 84.83–90.97%; good agreement with clinical laboratory results	30-fold serum dilution	Stable at neutral/basic pH; evaluated in biological assays at 37 °C	Operationally demonstrated in cell imaging and serum analysis; long-term storage stability not reported	33
2	Pyrene-appended thymine acetamide	—	—	Tested at pH 7.4	Reversible turn-on response; non-cytotoxic in Vero 76 cells; long-term stability not reported	34
3	Naphthyridine-based receptor	Serum: 96–97%; urine: 93–98.3%; close agreement with clinical methods	20-fold dilution	Effective at pH 7.4 and physiological temperature	Chemically stable and non-cytotoxic up to 100 µM; storage stability not reported	35
4	Inkjet-printed 1,8-naphthalimide PET chemosensor	Qualitatively compared with commercial UA strips and a ferric chloride/potassium ferricyanide assay	No dilution	—	Paper strips stored at 4 °C in the dark; quantitative storage stability not reported	36
5	1,8-Naphthalimide turn-on fluorescent chemosensor	—	—	pH 6–8; optimum enzyme-recognition temperature: 30–40 °C	Stable under continuous UV irradiation for 12 h; stable response after 25 min incubation	37
6	Rhodamine–naphthyridine fluorescent probe (R1)	Serum recovery: 84.83–90.97%; compared with clinical laboratory measurements	30-fold dilution	pH 1–14	Stable performance over a broad pH range; long-term stability not reported	38
7	Cu^2+^@MIL-91(Al : Eu) composite	429 µmol L^−1^ measured for 420 µmol L^−1^ spiked urine	—	pH 4–8; thermally stable to 473 K	Stable in water for 24 h to 3 days; reusable for four cycles	39
8	Coordination polymers P1–CP3	Recovery: 93.1–103.6%; RSD: 1.9–3.7%	100-fold dilution	pH 3–10; thermally stable to 356–360 °C	Stable in water for 24 h; reusable for six cycles	40
9	2-Picolinic-acid-functionalized UiO-66-NH_2_ (UiO-PSM)	Recovery: 96.94–104.58%; calibration *R*^2^ = 0.9986	—	pH 4–10; thermally stable to 264–478 °C	Stable for 2 weeks; fluorescence stable under UV irradiation for 60 min; reusable for four cycles	41
10	BSA-Au/Ag nanoclusters/HRP/OPD ratiometric probe	Consistent with an AU2700 automatic biochemical analyser	10-fold serum dilution	Reaction system: pH 6.0; 37 °C	Signal plateau after 45 min; long-term storage stability not reported	43
11	Cu^2+^@Tb-MOF fluorescent probe	—	—	pH 3–9; thermally stable to 400 °C	Water-tolerant for 24 h; reusable for four cycles	44
12	N,P–Co-doped carbon dots/Au@Ag nanoparticles	Urine recovery: 103–109%; close to official values	Deproteinisation followed by dilution	Optimum pH 11.5; optimum temperature: 50 °C	—	45
13	PVP-Cu nanoclusters/uricase	Urine recovery: 96.86–98.12%	—	Optimum pH 7.5	—	13
14	N-Doped carbon dots/Cr(vi) nanocomposite	—	—	—	Chemical and aqueous stability reported; quantitative data not provided	46
15	Zn-CD@Eu ratiometric FRET nanoprobe	UA recovery: 97.80–106.15%; RSD ≤ 2.96%	200-fold dilution of serum/sweat samples	pH 4–9	Fluorescence unchanged after 12 months; no measurable signal fluctuation over 1 h	47
16	Ln^3+^@UiO-66-(COOH)_2_ MOF	Theoretical study	—	—	—	48
17	Tb-based luminescent MOF sensor	—	—	—	—	49
18	Cd(ii)-MOF luminescent chemosensor	—	—	—	—	50
19	Eu/Zr-MOF ratiometric fluorescent sensor	—	30 mg MOF dispersed in 3.00 mL ethyl acetate	Room temperature	—	52
20	ssDNA/SWCNT corona-phase molecular-recognition sensor	HPLC used as the reference method	60-fold dilution	pH 4–8; room temperature	Storage stability up to 15 days	53
21	HAuCl_4_/TMB colorimetric probe	Recovery: 96.0–104.57%; calibration *R*^2^ = 0.9949–0.9954	1000-fold dilution	pH 3–6; 20 ± 2 °C	—	54
22	MoS_2_ nanoflakes/uricase/TMB–H_2_O_2_ sensor	Serum recovery >94.54%	Centrifugation followed by dilution in Britton–Robinson buffer	pH 8.5; 35 °C	—	55
23	Uricase/Cu^2+^/TMB colorimetric sensor	Urine recovery: 94.4–100.7%; mean relative error: 4.9%	Centrifugation and dilution; 120-fold effective dilution in recovery experiments	Uricase reaction: pH 8.5, 35 °C; Cu^2+^/TMB oxidation: pH 3.5, 45 °C	—	56
24	Ni@MnO_2_ oxidase-mimetic nanozyme	Serum recovery: 97.7–102.8%; good agreement with clinical values	—	Optimum pH 4.4; 30 °C	—	57
25	Ionic-liquid-capped Ni nanoparticles	Serum recovery: 101.15–102.18%	—	Optimum sensing pH 7.5	—	58
26	Cu-doped biochar nanozyme	—	—	Optimum pH 6.0	—	59
27	Fe_4_Ni nanoflowers	Standard solutions: 91.3–103.8%; serum: 90.6–111.9%	No dilution	Optimum pH 3.5; stable at 25–40 °C	Retained >90% activity after 7 days in ethanol; powder stable for 40 days at 4 °C	60
28	AgNPs@MCA	Urine recovery: 96.1–102.2%; RSD <4%	Centrifugation, ascorbate oxidase treatment, filtration and 50–100-fold dilution	Optimum pH 4.0	Three-batch reproducibility: RSD = 3.69%; stable for 50 days, RSD = 3.01%	61
29	Triangular Ag nanoprisms	Tested using serum from healthy and hyperuricaemic individuals	Five-fold serum dilution	—	Similar SPR spectra for three independently prepared batches	62
30	Uricase-coupled Ag nanoprisms	Serum recovery: 95.4–101.8%; RSD ≤ 3.1%; compared with a biochemical analyser	Two-fold serum dilution	Stable in neutral medium; performance decreased above 40 °C	No detectable spectral change after 1 h; long-term stability not reported	63
31	N-CQDs/Ag triangular nanoprisms dual-read sensor	Urine recovery: 93.4–105.6% (fluorometric) and 94.9–103.5% (colorimetric)	Urine analysed against a commercial UA kit	Borate buffer, pH 8.5	Stable and homogeneous for 1 h	64
32	N-CQDs/Ag triangular nanoprisms	Urine recovery: 93.4–105.6% (fluorometric) and 94.9–103.5% (colorimetric)	—	Fluorescence minimally affected by pH, ionic strength and time	Long-term stability not reported	65

#### 2.2.2 Chromogenic sensors based on nanozymes

The sensing platform exhibits good sensitivity, selectivity, and rapid response, rendering it suitable for the analysis of biological samples in practical applications. Furthermore, the method offers a straightforward and cost-effective alternative for point-of-care diagnostics, especially in resource-constrained environments. Overall, this approach presents a promising strategy for convenient and reliable monitoring of uric acid in clinical and biomedical contexts.

Zhang and co-workers developed a selective and sensitive colorimetric sensor system for uric acid (UA) utilizing 3,3′,5,5′-tetramethylbenzidine (TMB; 22; Fig. 22).^56^ The interaction between 3,3′,5,5′ tetramethylbenzidine (TMB) and hydrogen peroxide (H_2_O_2_) leads to a color change, a process frequently employed for colorimetric detection of various biomolecules. In this study, the researchers devised a method in which the enzyme uricase generates H_2_O_2_ from uric acid (UA), and the resulting H_2_O_2_ subsequently oxidizes TMB in the presence of Cu^2+^, leading to a visible color transition from colorless to blue. UV-vis spectroscopy identified a new absorbance peak around 650 nm, confirming UA detection by the TMB-based assay. A linear response to UA was observed over the concentration range of 1–100 µM, with a detection limit of 0.64 µM. The developed colorimetric sensor demonstrated reliable detection of the target analyte in urine samples, achieving recovery rates exceeding 94.0%. Additionally, the results showed strong correlation with those obtained from a bio-analyzer, with a mean relative error of 4.9%. The biosensor, which incorporates uricase, exhibited high selectivity and sensitivity for UA, without the need for sample pre-treatment. Consequently, the developed UA biosensor represents a promising platform for biochemical and clinical applications, while expanding the use of the Cu^2+^-catalyzed TMB–H_2_O_2_ reaction system in recognition-based sensing.

**Fig. 22:**
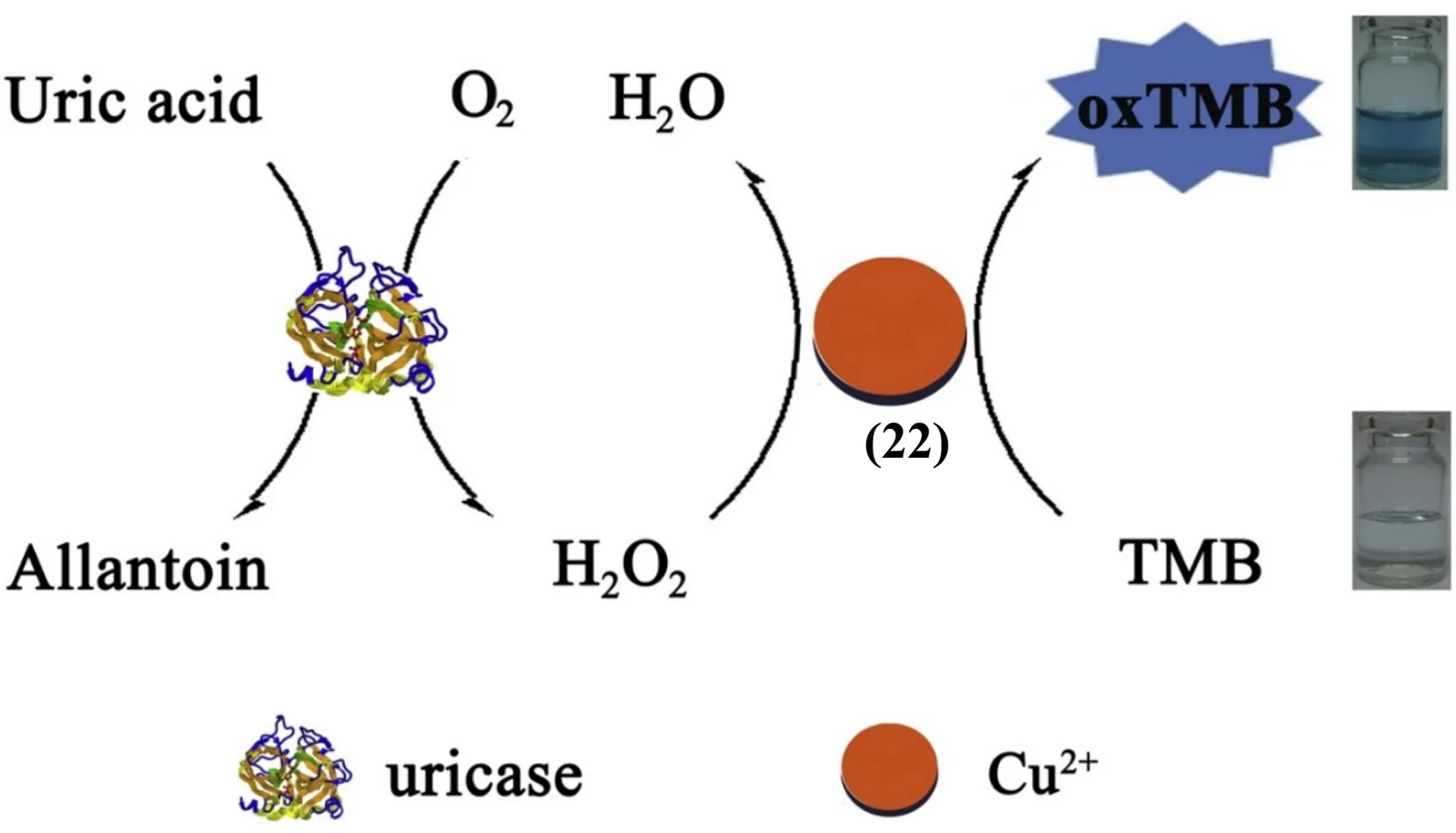
Schematic diagram of colorimetric uric acid sensor by utilizing uricase and Cu^2+^-catalyzed (22) (TMB) oxidation (this figure has been adapted from ref. 56 with permission from Elsevier, copyright 2017).

In pursuit of an efficient, cost effective and nonenzymetic method for the selective recognition and quantification of UA, Pal group employed 3,3′,5,5′-tetramethylbenzidine (TMB) and Ni@MnO_2_ hybrid nanomaterial (23; Fig. 23).^57^ This study introduced the first instance of an ultralong porous core–shell Ni@MnO_2_ hybrid nanomaterial synthesized through a straightforward redox transformation involving prickly nickel nanowires and KMnO_4_ solution. The formation mechanism was clarified using time-dependent FESEM and TEM characterization. The resultant Ni@MnO_2_ nanostructure exhibited intrinsic oxidase-like properties, enabling selective nonenzymatic detection of uric acid in serum samples. The material catalyzed the oxidation of TMB from a colorless state to a blue-colored product, which was subsequently decolorized in the presence of uric acid. Utilizing this mechanism, a sensitive colorimetric assay for UA was developed, demonstrating a detection limit of 0.24 µM and a linear range between 1 and 40 µM in real serum samples. The enhanced oxidase-mimicking activity was attributed to the large surface area and lattice oxygen of MnO_2_. The authors proposed that the developed approach holds strong potential for clinical UA monitoring.

**Fig. 23:**
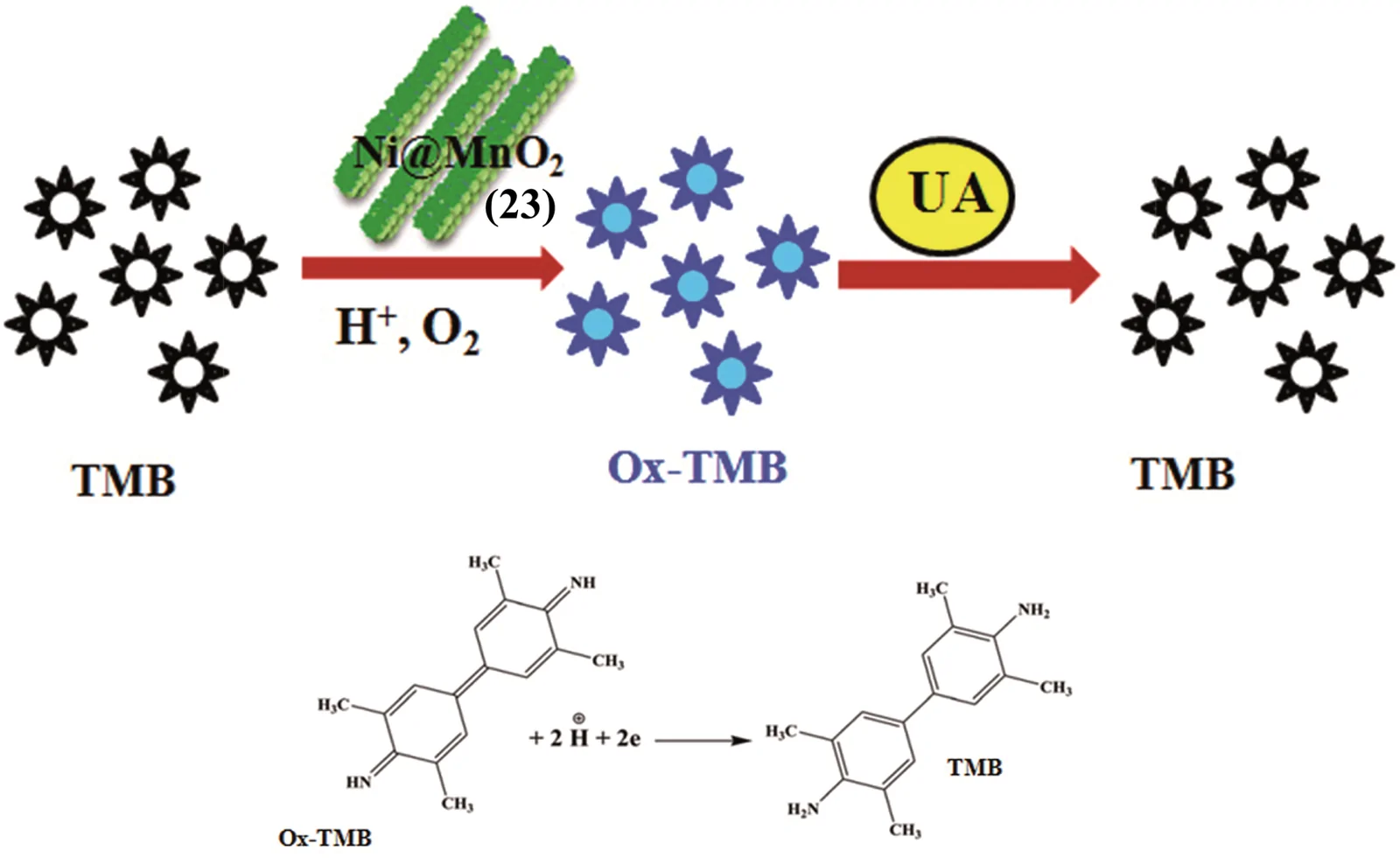
Schematic representation of colorimetric sensor for UA by using TMB and porous Ni@MnO_2_ (23) hybrid nanomaterial (this figure has been adapted from ref. 57 with permission from Royal Society of Chemistry, copyright 2016).

Nishan and colleagues developed a biosensor to detect uric acid (UA) in test samples, utilizing 1-*H*-3-methylimidazolium acetate (ionic liquid, IL)-capped nickel nanoparticles (NiNPs) (24; Fig. 24).^58^ The IL-capped NiNPs and their precursors underwent analysis through various instrumental techniques, including Fourier transform infrared spectroscopy, scanning electron microscopy, and X-ray diffraction.

**Fig. 24:**
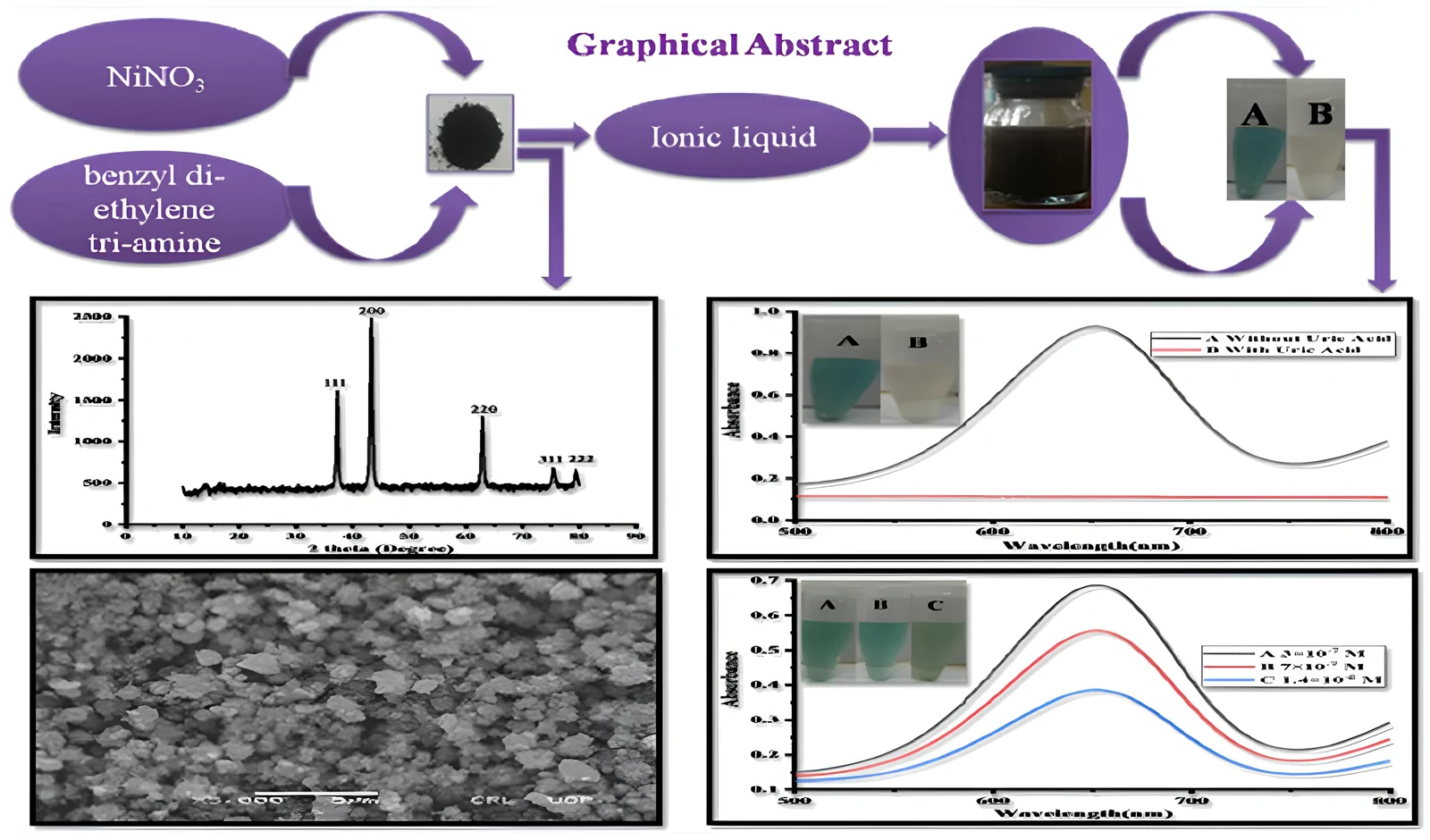
Schematic representation of the synthesis of ionic liquid capped NiNPs (24) and the recognition of UA using this system (this figure has been adapted from ref. 58 with permission from American Chemical Society, copyright 2022).

The IL-capped nickel nanoparticle solution, characterized by its green appearance, exhibited absorption around 650 nm. Upon the addition of UA, the green solution turned colorless, with the absorption spectra demonstrating complete quenching. The sensing experiment was optimized conditions, covering a UA concentration range from 1 × 10^−8^ to 2.4 × 10^−6^ M. The sensing material displayed significant peroxidase-like catalytic activity over a broad linear range. An increase in UA concentrations led to a reduction in absorbance intensity, as evidenced by suppression of the UV–vis absorption band at 652 nm. The biosensor achieved excellent linear detection from 1 × 10^−8^ to 2.4 × 10^−6^ M, with a limit of quantification (LOQ) of 4.3 × 10^−7^ M, limit of detection (LOD) of 1.30 × 10^−7^ M, and a high *R*^2^ value of 0.9994. The device exhibited high sensitivity to UA in the presence of various potential interferents and maintained its sensing response in human serum. The interference study included ethanol, K^+^, urea, dopamine, methanol, Ca^2+^, ascorbic acid, and glutathione, each at a concentration of 2.40 × 10^−6^ M. However, the selectivity assessment is incomplete, as significant physiologically relevant interferents such as xanthine, hypoxanthine, and creatinine were not evaluated. Additionally, competitive interference experiments involving mixtures of multiple coexisting species were not conducted. Consequently, although IL-coated NiNPs appear promising as a sensing platform for UA determination in various sample matrices, more comprehensive interference studies are necessary to establish their practical selectivity under physiologically relevant conditions.

The Nishan group developed a citric-acid-functionalized Cu-doped biochar nanozyme (CA–Cu@BC) that mimics peroxidase activity and enables redox-mediated colorimetric sensing of uric acid (25; Fig. 25).^59^ The characterizations of 25 was conducted using various analytical techniques. The sensor showed peroxidase-mimicking behavior, which generates hydroxyl radicals on the nanozyme surface to oxidize TMB with H_2_O_2_ into its blue-green state (*λ*_max_ = 652 nm). Introducing uric acid (UA) reverses this color change by reducing TMB_ox_ back to colorless TMB as UA oxidizes into allantoin. Several physical and experimental parameters including pH, reaction duration, IL content, TMB concentration, and UA concentration, were systematically optimized to enhance sensor performance. Under optimized conditions (2 mg of the nanozyme, pH 6, time 150 seconds), the biosensor demonstrated a linear response within the range of 1–90 µM, with an LOD of 0.17 µM, an LOQ of 0.58 µM, and a high regression coefficient (*R*^2^) of 0.997. Selectivity of the sensor was evaluated against Zn^2+^, Mg^2+^, Ca^2+^, glucose, dopamine, ascorbic acid and their mixture, with the authors reporting negligible interference. However, the group missed some important competitive species such as glutathione, dopamine, xanthine, hypoxanthine and creatinine. The method was further applied to diluted urine samples, demonstrating practical applicability for UA determination.

**Fig. 25:**
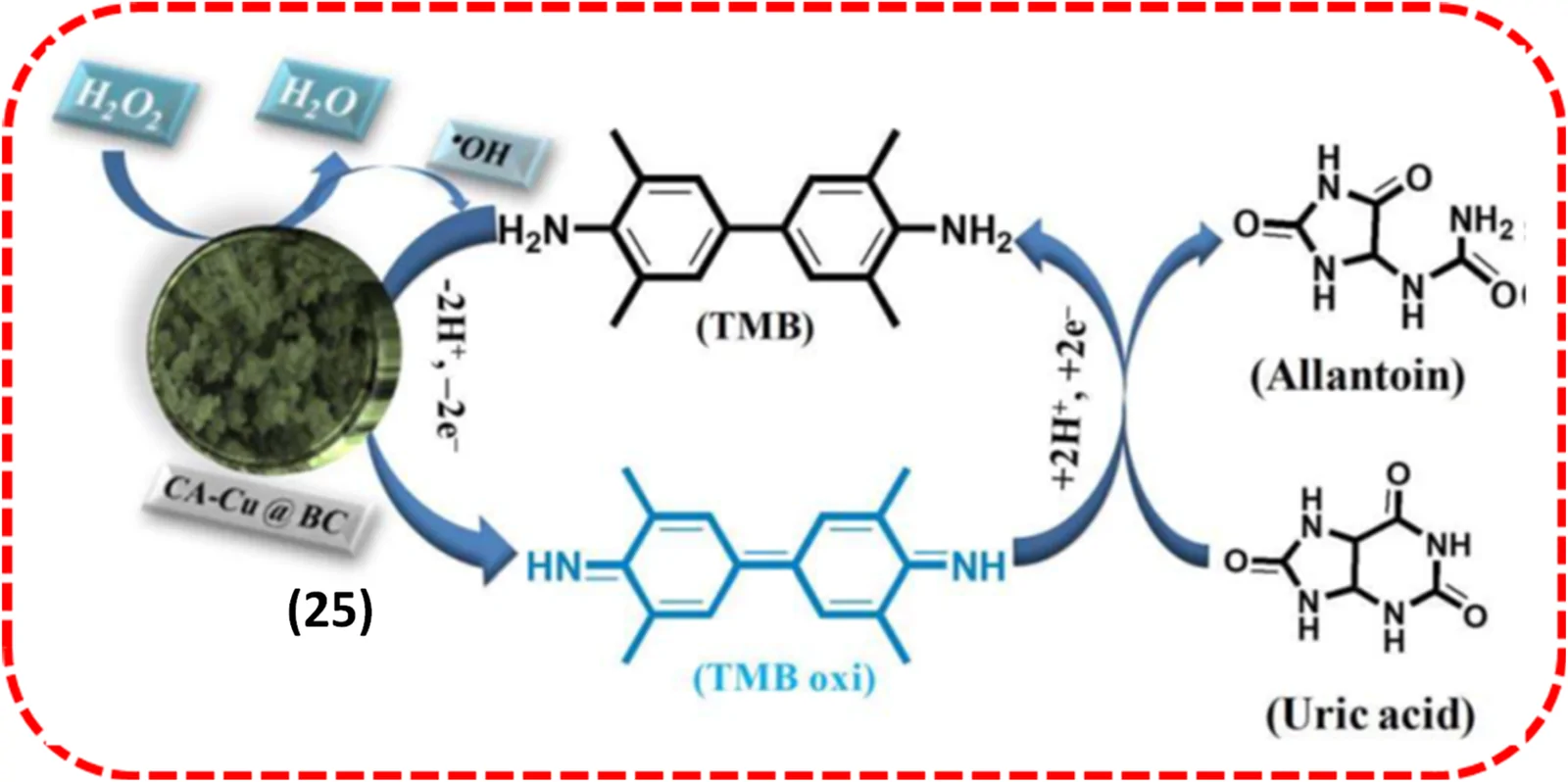
Schematic representation of a colorimetric sensor for UA by using ionic liquid capped NiNPs (25) (adapted from ref. 59). (this figure has been adapted from ref. 59 with permission from Royal Society of Chemistry, copyright 2024).

Qin and co-workers have developed a series of unique iron–nickel bimetallic nanoflowers, Fe_4_Ni-NFs (26; Fig. 26), which demonstrate significant peroxidase (POD)-like catalytic activity.^60^ Among the synthesized materials, the Fe_4_Ni-NFs with the highest catalytic efficiency were utilized to were utilized to create a straightforward point-of-care (POC) colorimetric platform for detecting uric acid (UA). This strategy relied on uricase-mediated conversion of UA into H_2_O_2_, which subsequently facilitates the oxidation of chromogenic substrates catalyzed by Fe_4_Ni-NFs, resulting in observable color changes. The Fe_4_Ni nanoflowers displayed excellent POD-mimicking catalytic activity in the oxidation of TMB, ABTS, and OPD in the presence of H_2_O_2_. Distinct absorbance peaks were observed at 652 nm for TMB, 420 nm for ABTS, and 450 nm for OPD, accompanied by progressive color changes from colorless to blue, green, and orange–yellow, respectively, as concentration of H_2_O_2_ increased. The developed sensing platform enabled rapid and visual determination of UA in serum, with a linear detection range of 2–500 µM and an LOD of 1.13 µM. Furthermore, the system was effectively employed to monitor serum UA levels in hyperuricemic rats undergoing treatment with allopurinol and benzbromarone, demonstrating its potential for rapid pharmacodynamic assessment. The findings underscore the broad applicability of Fe_4_Ni-NFs in precision therapeutics and drug development, while also offering valuable insights into the catalytic mechanisms of bimetallic nanozymes for the diagnosis and treatment of hyperuricemia.

**Fig. 26:**
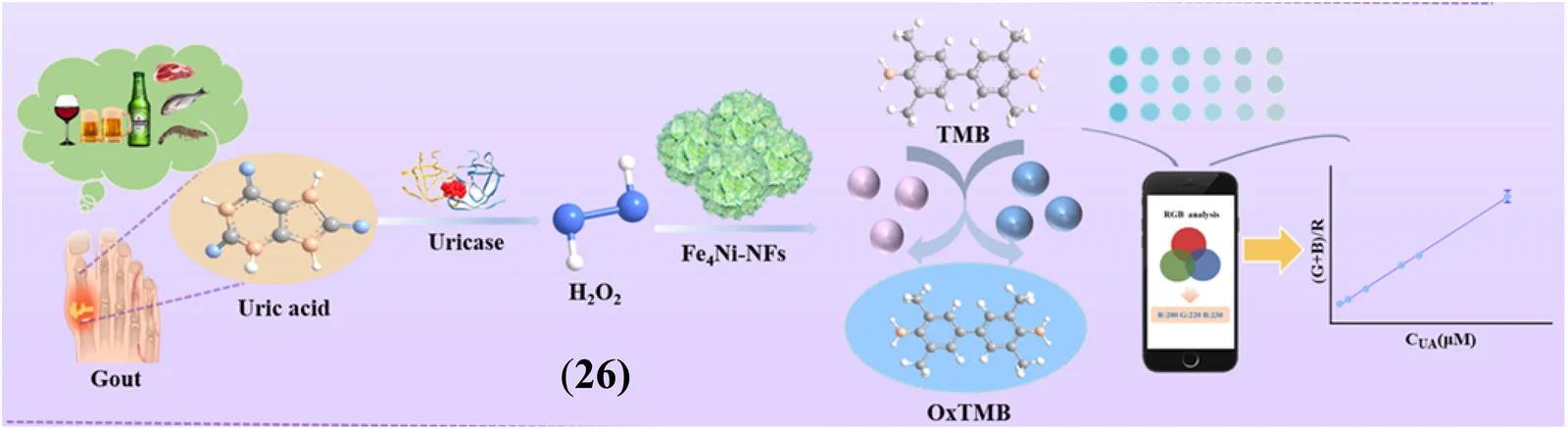
Schematic diagram illustrating the peroxidase-like catalytic activity of Fe_4_Ni-NFs nanoflowers (26) for ultrasensitive uric acid detection and hyperuricaemia therapy evaluation. (this figure has been adapted from ref. 60 with permission from Royal Society of Chemistry, copyright 2026).

Dongbo and co-workers developed AgNPs@MCA (27; Fig. 27) nanozymes exhibiting significant oxidase-mimicking activity.^61^ Surface modification of silver nanoparticles with MCA notably enhanced their catalytic efficiency by altering the reaction pathway and facilitating oxygen activation. The nanozymes generated reactive oxygen species, including ·OH and ^1^O_2_, which oxidized TMB, resulting in a distinct colorimetric response. Utilizing this mechanism, the researchers devised a straightforward and highly sensitive colorimetric strategy for detecting uric acid (UA). As UA scavenges these reactive oxygen species, the color intensity diminished proportionally with increasing UA concentration, allowing for quantitative analysis. The sensing platform demonstrated a linear response within the 20–100 µM range, with a detection limit of 1 µM. Furthermore, the method was successfully applied to UA determination in urine samples, indicating its potential for early gout diagnosis.

**Fig. 27:**
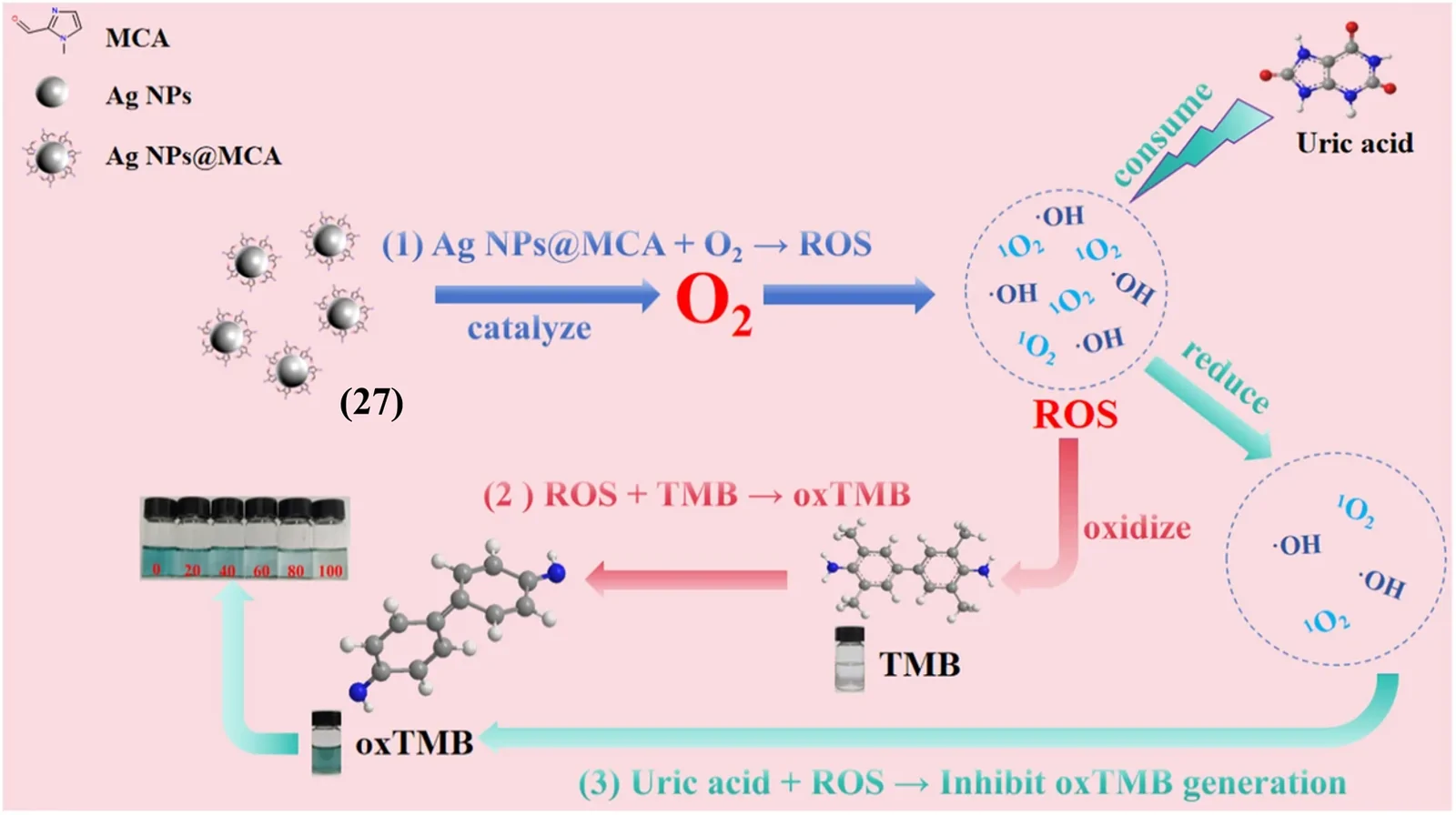
Schematic diagram of the principle of uric acid detection by the probe 27 (this figure has been adapted from ref. 61 with permission from Springer Nature, copyright 2025).

#### 2.2.3 Nanoprism-based colorimetric sensor

Xia and collaborators proposed a colorimetric sensing strategy for blood uric acid (UA) utilizing unmodified silver (Ag) nanoprisms, employing a facet-dependent binding/etching mechanism as the signal readout system (28; Fig. 28).^62^ In the absence of the target molecule, triangular Ag nanoprisms undergo etching induced by H_2_O_2_ at the (110) facets, resulting in the formation of round nanodiscs accompanied and an associated surface plasmon resonance (SPR) blue shift of less than 160 nm. Conversely, UA molecules pre-adsorbed onto the side facets of the nanoprisms exhibit strong binding, thereby inhibiting the etching process.

**Fig. 28:**
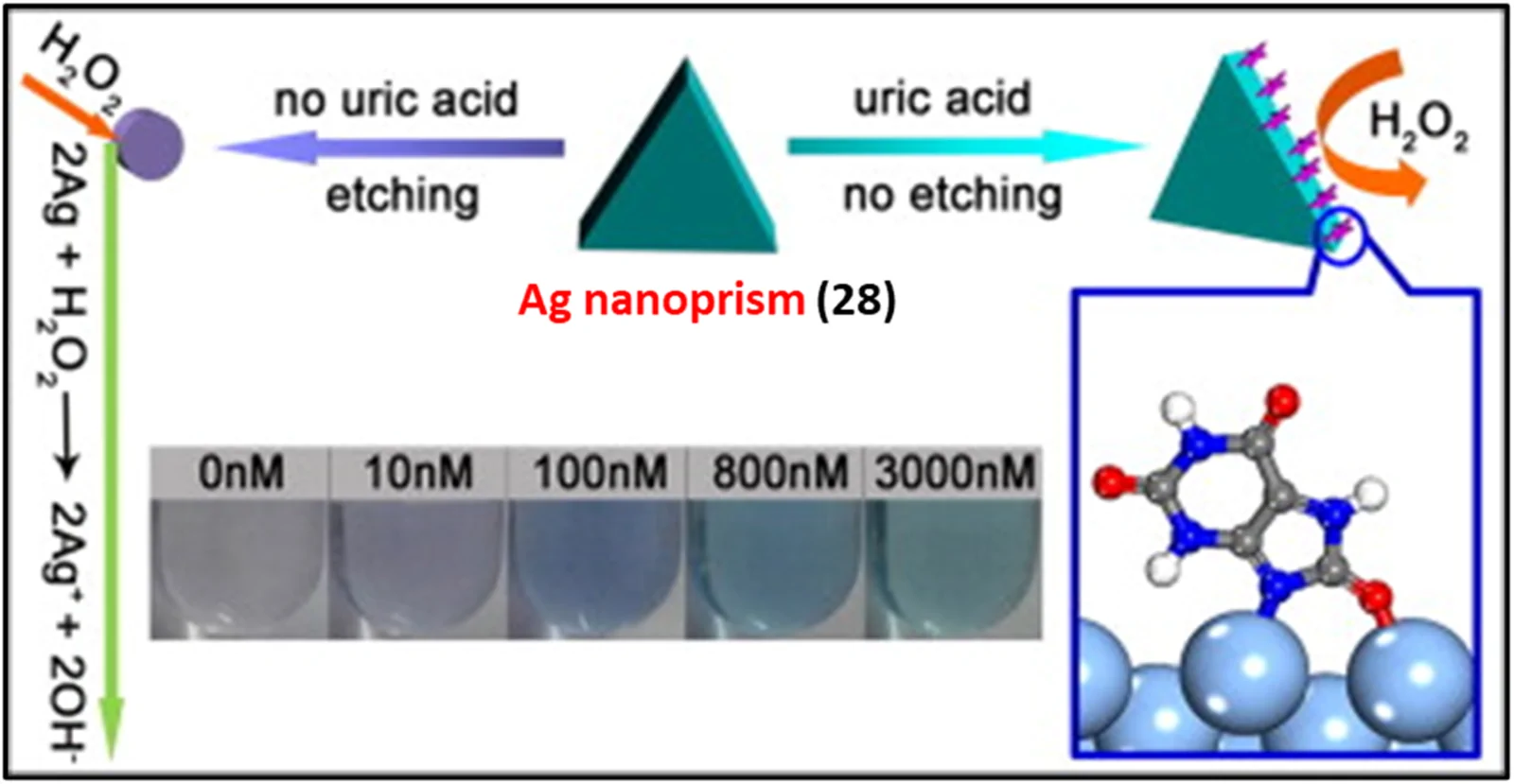
Schematic representation of the recognition of the UA by the Ag nanoprism (28) (this figure has been adapted from ref. 62 with permission from Elsevier, copyright 2014).

An inverse correlation was identified between the SPR blue shift and UA concentration, facilitating the quantitative determination of UA within a range of 10–3000 nM with a detection limit as low as 10 nM. The exceptional sensitivity of the sensing platform is attributed to three primary factors: (a) the selective etching of Ag nanoprism side facets by H_2_O_2_, (b) the preferential binding of UA to the (110) facets of the nanoprisms, and (c) the ultrathin plate-like structure (∼5 nm), which minimizes the available side area. Additionally, the assay allows for straightforward visual differentiation between hyperuricemic and healthy samples. Consequently, this simple and economical colorimetric sensor holds significant diagnostic potential.

Zhang and his team utilized the morphological transition of Ag nanoprisms to develop an effective method for colorimetric uric acid detection. In presence of uricase, UA was oxidised to form H_2_O_2_. The etching by H_2_O_2_ altered the shape of Ag nanoprisms (29; Fig. 29)^63^ from triangle to round, resulting in a blue shift of over 120 nm in the surface plasmon resonance (SPR) spectrum band. This color change, observable by the naked eye from pale blue to purple, correlates with variations in UA content. A strong linear relationship was established between the SPR blue shift of Ag nanoprisms and UA concentrations ranging from 1 to 40 µM, with a detection limit as low as 0.7 µM. Various potential serum interferents, such as ascorbic acid, glucose, dopamine, hypoxanthine, and amino acids, did not significantly impact the sensing response (Fig. 29). The entire assay required was completed within one hour after the addition of serum samples to the Ag nanoprism–uricase sensing device. The proposed sensing system effectively quantified UA in human serum samples, achieving recoveries exceeding 95%, and the results closely aligned with those from a standard biochemistry analyser, yielding an average relative error of 5.1%. The Ag nanoprism–uricase sensor offers rapid analysis, low-cost instrumentation, high selectivity, and high sensitivity without necessitating sample pretreatment, undercrossing its potential applicability in clinical diagnostics.

**Fig. 29:**
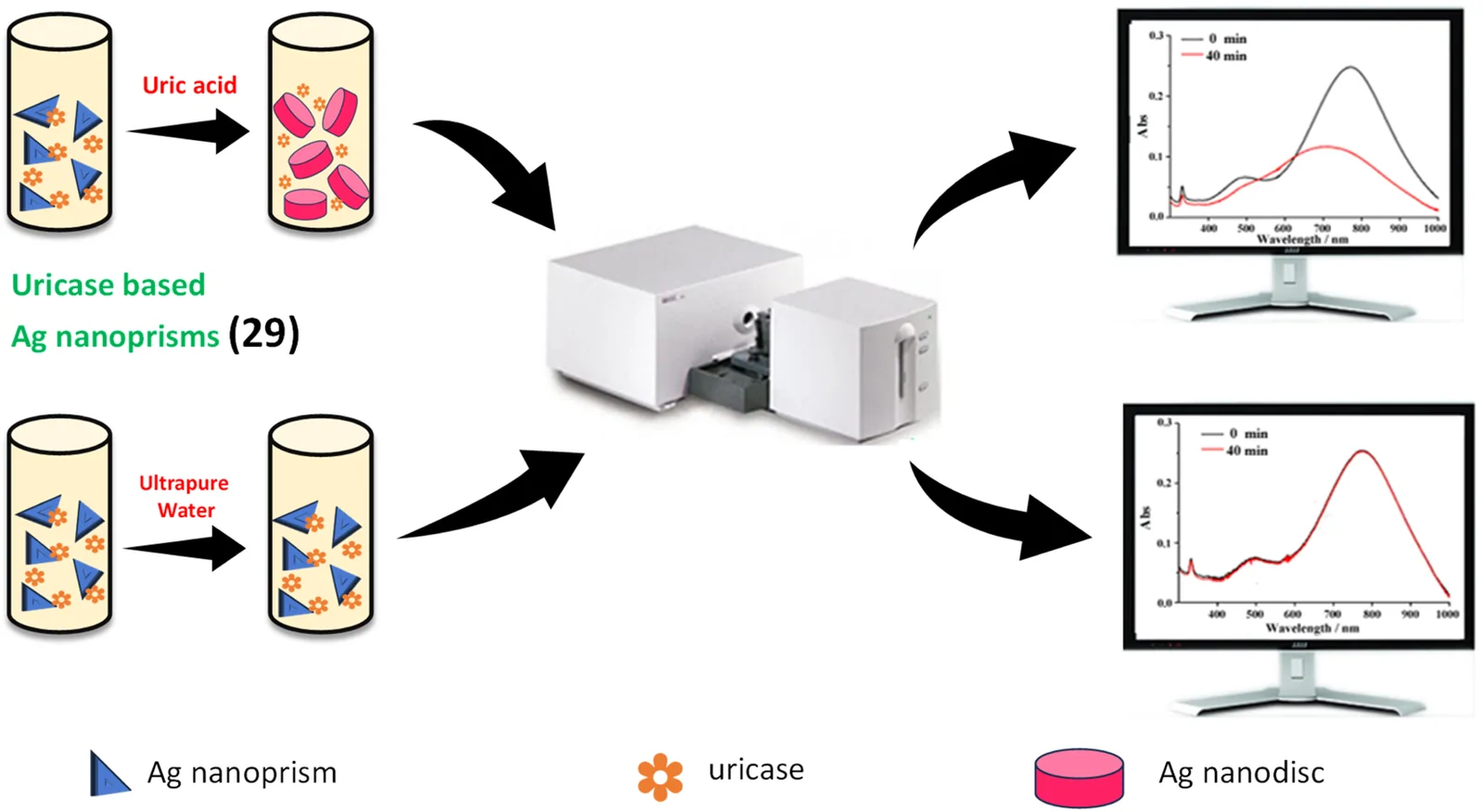
Schematic diagram of colorimetric sensing of uric acid based on uricase-catalyzed etching of Ag nanoprisms (29) (this figure has been adapted from ref. 63 with permission from Elsevier, copyright 2015).

Wang *et al.* have developed a dual-mode, non-enzymatic uric acid (UA) sensor that integrates nitrogen-doped carbon quantum dots (N-CQDs, exhibiting blue fluorescence at 440 nm upon excitation at 340 nm) with silver triangular nanoprisms (AgTNPs, with a surface plasmon resonance band at 683 nm). This configuration facilitates simultaneous fluorometric and colorimetric detection. Initially, the AgTNPs (30; Fig. 30) quench the fluorescence of N-CQDs through an inner filter effect due to spectral overlap, rather than electrostatic interaction, as both components possess negatively charged surfaces.^64^ The sensing mechanism is based on the facet-dependent etching of AgTNPs by H_2_O_2_, which preferentially targets the high-energy (110) facets and sharp tips of the nanoprisms, transforming them into rounded nanodiscs and reducing the absorbance at 683 nm. UA exhibits strong binding to the (110) facets, thereby protecting the nanoprisms from etching. Consequently, an increase in UA concentration preserves the triangular morphology, enhances the 683 nm absorbance, and increases fluorescence quenching. Under optimized conditions—boric acid buffer at approximately pH 8.5, 15 minute incubation, and a 900 µL UA sample volume—the N-CQD fluorescence remained stable across variations in pH, ionic strength, and time, with no significant signal deterioration observed over one hour. Analytically, the fluorometric mode demonstrated a linear range of 1–42 µM with a limit of detection (LOD) of 0.2 µM (*R*^2^ = 0.997), while the colorimetric mode exhibited a broader linear range of 0.1–45 µM with an LOD of 50 nM (*R*^2^ = 0.993). This was accompanied by a visible color change from light blue to navy blue, enabling detection without instrumentation. Selectivity testing against a wide array of urinary constituents (K^+^, Na^+^, Mg^2+^, Zn^2+^, amino acids, ascorbic acid, glucose, dopamine, caffeine, theophylline, adenine, guanine, hypoxanthine, xanthine, and theobromine) at 25 µM showed minimal interference (Fig. 31). Validation in human urine against a commercial UA kit yielded recoveries of 93.4–105.6% (fluorometric) and 94.9–103.5% (colorimetric). However, the authors noted that the probe is not suitable for samples with complex matrices, despite its successful application in urine.

**Fig. 30:**
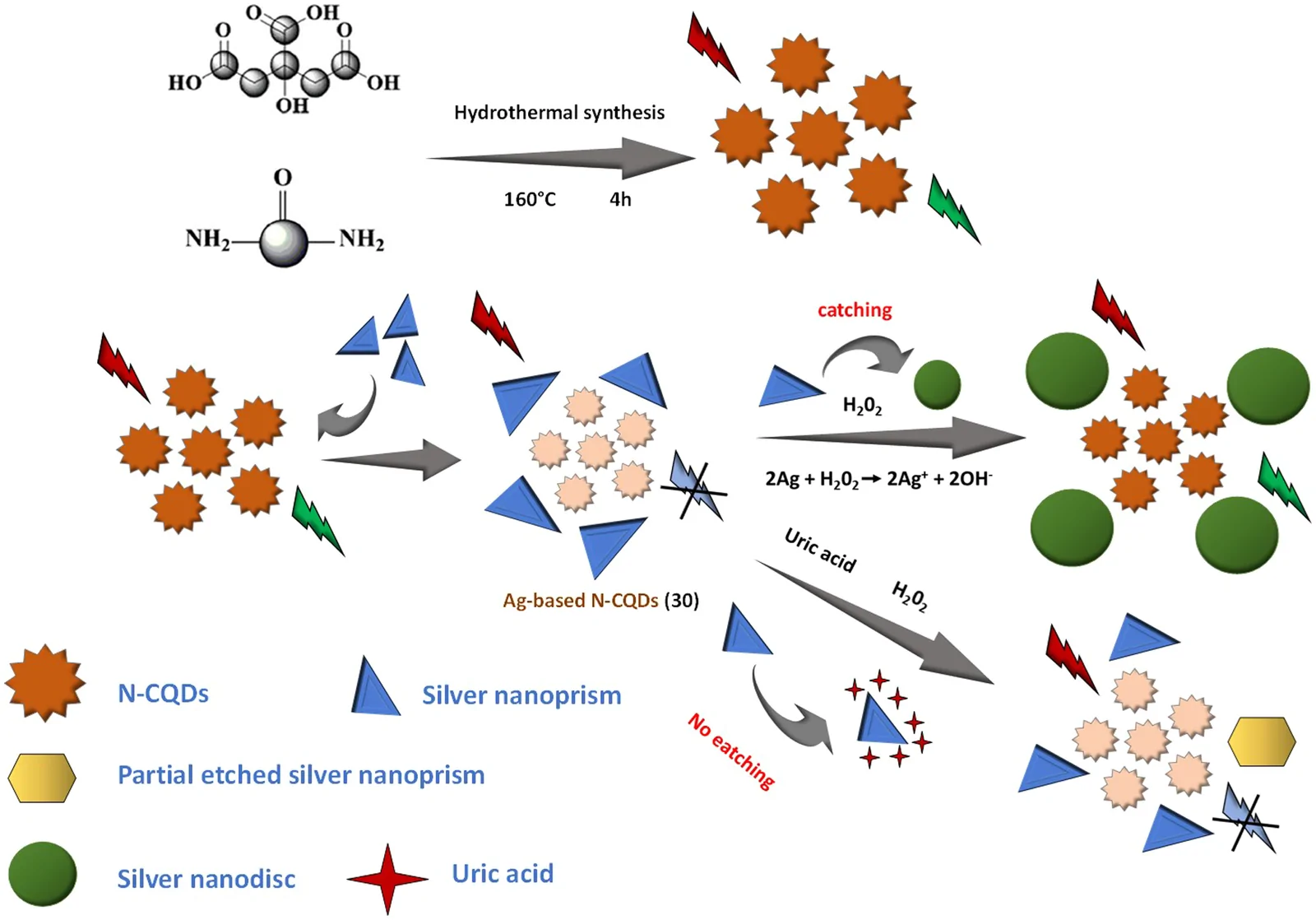
Schematic representation of uric acid detection by the probe 30.

**Fig. 31:**
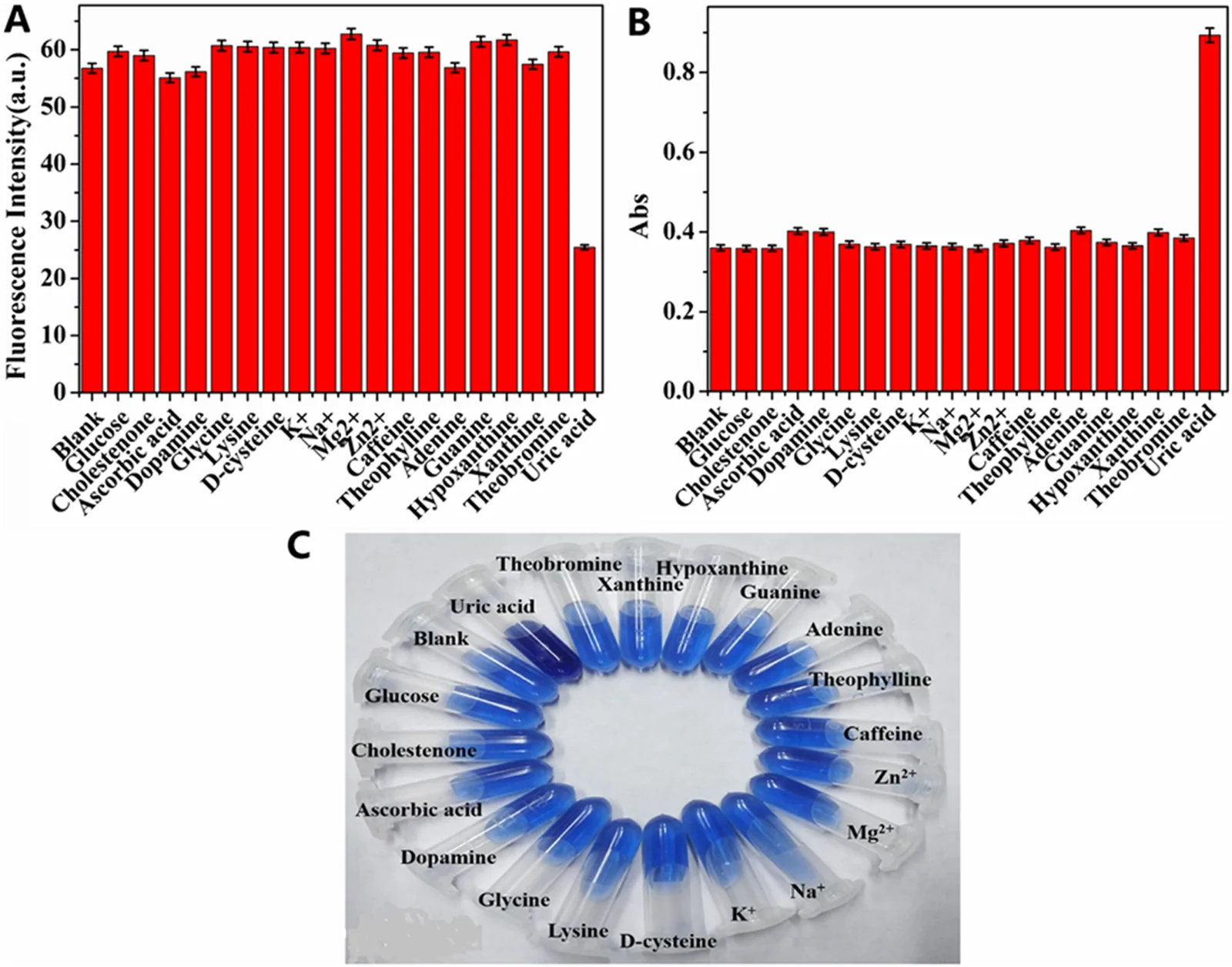
(A) The fluorescence intensity of N-CQDs/AgTNPs in the presence of UA or various ions and molecules at the concentration of 25 µM. The data is acquired at excitation/emission peaks of 340/440 nm. (B) Absorbance response of the N-CQDs/AgTNPs probe in the presence of UA or various metal ions and molecules at the concentration of 25 µM. The data is acquired at absorption peaks of 683 nm. (C) The photograph is the colorimetric response for various substances (this figure has been adapted from ref. 64 with permission from Springer Nature, copyright 2018).

## 3 Challenges and potential strategies

Despite the rapid advancement of fluorescent and colorimetric sensors for uric acid (UA) detection, these technologies predominantly remain in the experimental phase. They continue to encounter challenges related to selectivity, enzyme dependence, stability during application, device integration, and clinical testing.^65–67^ Enzyme-based assays, particularly those utilizing uricase, are still prevalent.^68–70^ However, issues such as enzyme degradation, inconsistent activity, limited shelf life, and a restricted functional range undermine their reliability and practicality for point-of-care or field applications.^71^

These challenges have spurred interest in enzyme-mimicking materials and direct, enzyme-free detection methods. Nevertheless, most synthetic receptors and nanozymes require extensive evaluation for long-term stability, batch-to-batch consistency, and efficacy in real biological samples. In direct detection strategies, the inherent reducing properties of UA facilitate signal generation. However, this method lacks specificity, as other biologically prevalent reducing agents, including dopamine, ascorbic acid, glutathione, and cysteine, along with chemically similar purine compounds, can interfere with signals or quench fluorescence.^72,73^

Although advancements such as engineered recognition sites, functionalized metal–organic frameworks (MOFs), ratiometric fluorescence, and dual-mode detection have enhanced the capacity to differentiate uric acid (UA) from interferents, selectivity is frequently assessed under simplified conditions or with unrealistically low levels of interferents. Consequently, the actual performance of these techniques under real-world competitive conditions remains uncertain. Imaging within biological tissue presents additional challenges, as many fluorescent probes depend on UV or visible light excitation, which induces tissue autofluorescence, restricts light penetration depth, and poses a risk of tissue damage. Furthermore, the dynamics of UA movement and distribution within cells over time are not well understood. Near-infrared (NIR-I/NIR-II) probes, two-photon excitation methods, and lanthanide-based MOFs present promising solutions; however, their safety in biological systems, resistance to photobleaching, and measurement accuracy require validation in realistic cell and animal models.

A significant gap in the current research is the infrequent integration of UA sensing technologies with wearable, automated, or connected devices, which could facilitate continuous monitoring in sweat, saliva, blood, or urine (Fig. 32).^74^ Future research should shift focus from merely achieving lower detection limits to prioritizing sensors that can endure complex biological matrices, function without enzymatic components, provide reproducible results, and demonstrate clinical validation, preferably in conjunction with wearable or portable detection platforms. Ultimately, the critical factor will be benchmarking these sensors against established clinical reference methods and verifying their reliability in real patient samples. This will determine whether the sensitivity achieved at the laboratory level can be effectively translated into point-of-care testing, personalized healthcare, and real-time medical applications.

**Fig. 32:**
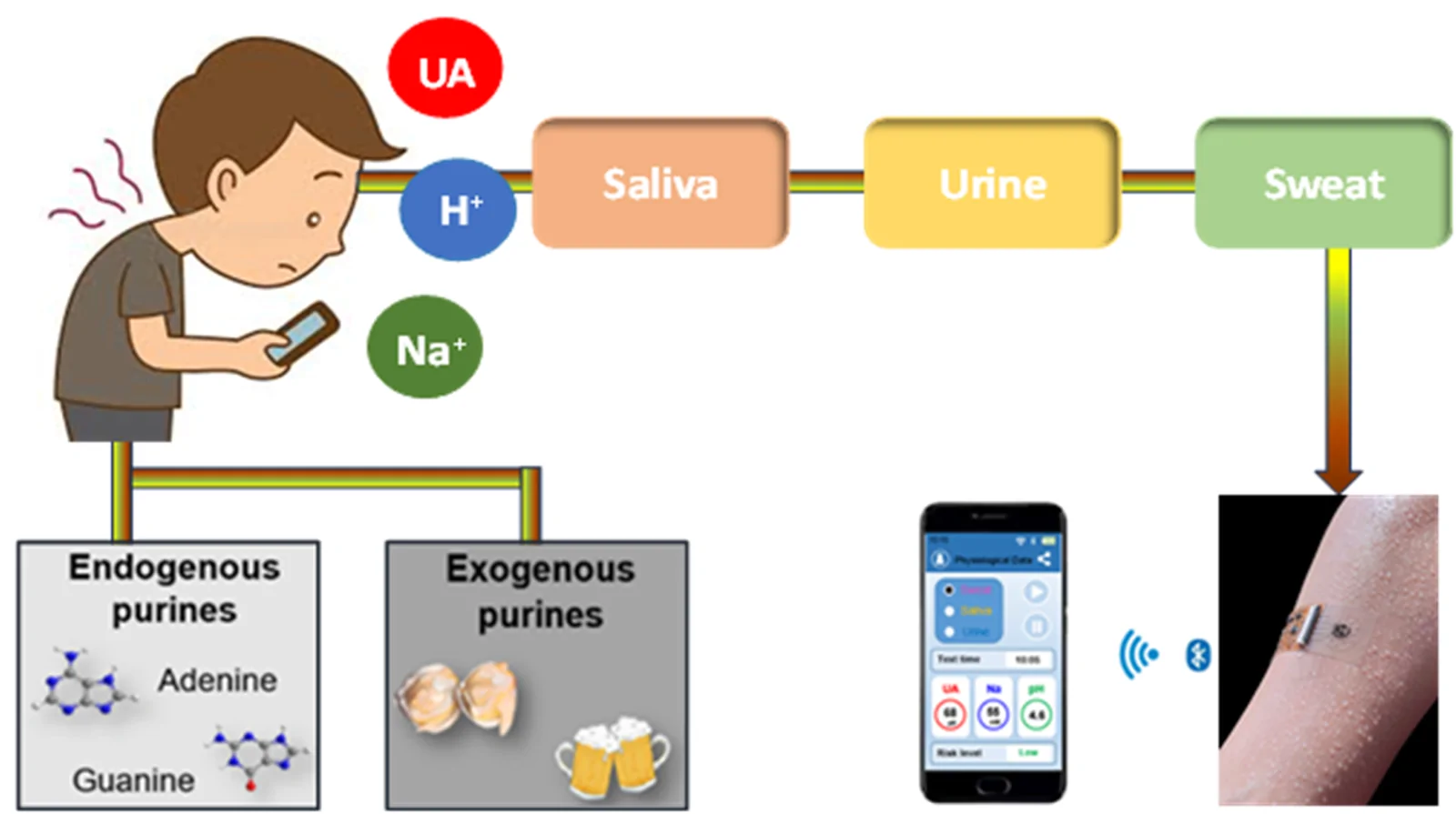
Illustration of the universal UA analysis in raw sweat, urine, or saliva (this figure has been adapted from ref. 74 with permission from American Chemical Society, copyright 2023).

## 4 Future directions

Future research should prioritize the translation of optical uric acid (UA) sensors from laboratory prototypes to wearable, portable, and clinically validated point-of-care diagnostic platforms, rather than solely focusing on achieving lower detection limits. The development of flexible microfluidic, paper-based, hydrogel, and stretchable devices could facilitate non-invasive or minimally invasive UA monitoring in biofluids such as sweat, saliva, tears, blood, and urine, thereby supporting the management of gout, hyperuricemia, and related metabolic or renal disorders. However, reliable analysis of these biofluids necessitates the control of matrix-dependent variables, including pH, dilution, secretion rate, and endogenous interferents, alongside validation against clinically relevant UA concentrations. Multiplexed sensor arrays capable of concurrently measuring UA, creatinine, glucose, urea, and electrolytes may offer greater diagnostic value than single-analyte assays. Additionally, chemometric and machine-learning approaches could enhance the interpretation of complex response patterns; however, their clinical utility requires extensive, diverse datasets and independent validation. Smartphone-assisted and portable optoelectronic readouts present practical avenues for decentralized testing, provided that variations in illumination, camera characteristics, sample volume, and image acquisition are addressed through standardized calibration and image-processing protocols. Similarly, the translation of quantum dots, metal nanoparticles, metal–organic frameworks (MOFs), and related nanomaterials necessitates systematic evaluation of biocompatibility, leaching, stability, and shelf-life in authentic biological matrices. Ultimately, these platforms must be benchmarked against established clinical reference methods in well-characterized patient cohorts, with rigorous assessment of accuracy, precision, recovery, interference, reproducibility, and batch-to-batch consistency. Therefore, the development of next-generation UA sensors should transition from prioritizing record analytical sensitivity to achieving clinically concordant, matrix-tolerant, reproducible, and standardized measurements in deployable formats, thereby enabling reliable disease monitoring and personalized preventive healthcare.

## 5 Conclusion

In this review provides a comprehensive summary and evaluation of various recognition strategies for the selective detection of uric acid (UA), with a particular focus on the interaction mechanisms between host molecules and UA analytes. The sensing systems are categorized based on the nature of host–guest interactions into hydrogen-bonding and other non-covalent interaction-based sensors, metal ion-coordinated systems such as MOFs and coordination polymers, and nanomaterial-assisted sensing platforms including nanoparticles, nanoprisms, and nitrogen-doped carbon dots. Significant advances have also been made in the recognition and imaging of UA in biological fluids, such as urine and blood serum, as well as in living cells. Numerous studies have demonstrated the successful design of sensor probes utilizing selective host–guest interaction strategies for UA sensing and imaging applications. Ongoing refinement of these sensing approaches anticipated to produce more efficient and highly selective systems with lower detection limits for real biological samples. Despite the substantial progress discussed in this review, several challenges persist. Each sensing platform possesses distinct advantages and limitations. Among the available approaches, fluorescent chemosensors based on small organic molecules exhibit particularly rapid and accurate responses toward UA. However, challenges related to cytotoxicity, pH-dependent sensitivity, and selectivity still require further improvement. Optical UA sensors also offer versatile opportunities for *in situ* and *in vivo* fluorescence imaging in live cells. Nonetheless, intracellular UA detection challenging due to of short excitation wavelengths and interference from tissue autofluorescence. Furthermore, monitoring UA distribution across different cellular compartments and body fluids continues to be a major challenge. Therefore, further efforts are needed to develop advanced recognition systems capable of precise intracellular localization and dynamic monitoring of UA fluctuations within various cellular regions. Such intracellular imaging studies may provide deeper insight into the biological roles and metabolic significance of UA. Overall, selective UA sensing in complex biological environments is expected to remain an active area of research, promoting the development of increasingly efficient and selective sensing strategies for distinguishing UA from other purine metabolites.

## Conflicts of interest

There are no conflicts to declare.

## Data Availability

No primary research results, software or code have been included and no new data were generated or analysed as part of this review.
